# Vitamin D attenuates the progression of pulmonary fibrosis via inhibiting thymidine kinase 1/PFKFB3-driven glycolysis

**DOI:** 10.1186/s12967-025-07298-1

**Published:** 2026-04-22

**Authors:** Huanyu Yang, Li Zhang, Mengjia Han, Keye Zhu, Xianghan Guo, Wenkang Yang, Qi Xu

**Affiliations:** 1 School of Public Health, Binzhou Medical University, NO 346, Guanhai Road, Yantai, Shandong Province 264003 China; 2 School of Health Management, Binzhou Medical University, Yantai, 264003 China; 3 Second School of Clinical Medicine, Binzhou Medical University, Yantai, 264003 China

**Keywords:** Silicosis, Vitamin D, Glycolysis, TK1, PFKFB3

## Abstract

**Background:**

Pulmonary fibrosis is characterized by the excessive deposition of extracellular matrix produced from myofibroblasts in the lungs. Enhanced glycolysis has been linked to fibroblast-myofibroblast transition (FMT) during pulmonary fibrogenesis, however, there is still a lack of pharmaceutical agents to target the metabolic dysregulation. Emerging evidence highlights Vitamin D’s anti-fibrotic potential, yet its role in modulating fibroblast glycolysis and pulmonary fibrosis remains unclear.

**Methods:**

MRC-5 cells and mouse primary lung fibroblasts treated with TGF-β1 combined with Vitamin D were used to explore the role of vitamin D on fibroblast activation and glycolysis in vitro. Silica and bleomycin-induced pulmonary fibrosis mouse model was established to evaluate the antifibrotic effects of Vitamin D and the glycolysis inhibitor 3-[3-pyridinyl]-1-[4-pyridinyl]-2-propen-1-one (3PO) in vivo. Cell metabolism assays were performed to determine the glycolytic rate. RNA sequencing was utilized to analyse the underlying molecular mechanisms by which Vitamin D affects fibroblast activation and glycolysis.

**Results:**

Transcriptomic analysis and subsequent experiments demonstrated that TK1 acts as a functional downstream effector of Vitamin D, and its expression was negatively regulated by Vitamin D. Knockdown of TK1 inhibited the profibrotic effects of TGF-β1 by reducing fibroblast glycolysis. Furthermore, 6-phosphofructo-2-kinase/fructose-2,6-bisphosphatase 3 (PFKFB3), a key driver of glycolysis, was identified as a target of TK1. Mechanistically, Vitamin D could reduce the expression of TK1, thereby partly blocking fibroblast glycolysis and activation by inhibiting PFKFB3. In a murine model of silica or bleomycin-induced fibrosis, both Vitamin D and 3PO treatment alleviated pulmonary fibrosis.

**Conclusions:**

Our findings revealed that Vitamin D could attenuate pulmonary fibrosis by blocking fibroblast glycolysis and FMT through the TK1-PFKFB3 pathway. Vitamin D supplementation or targeting fibroblast glycolysis could be promising treatment strategies for pulmonary fibrosis.

**Supplementary Information:**

The online version contains supplementary material available at 10.1186/s12967-025-07298-1.

## Graphical Abstract

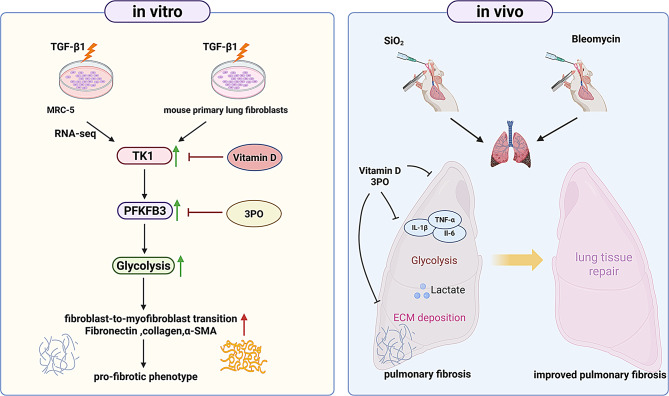

## Introduction

Pulmonary fibrosis, a chronic and progressive interstitial lung disease, is characterized by pathological fibrosis and abnormal scarring in pulmonary tissue with limited available drugs [1]. Chronic lung inflammation initiated by infectious agents, environmental toxins, or various medical interventions has been shown to contribute to the initiation and progression of pulmonary fibrosis [2]. Silicosis, a specific occupational form of pulmonary fibrosis resulting from inhaling crystalline silica, causes significant morbidity and mortality globally, particularly in mining, construction, and artificial stone processing [3, 4]. Like other fibrotic conditions, the characteristics of silicosis include damage to alveolar epithelial cells, epithelial-mesenchymal transition (EMT), fibroblast overgrowth, and excessive deposition of extracellular matrix (ECM) proteins [5]. Therefore, targeting these pathological alterations represents a promising approach for treating pulmonary fibrosis.

Fibroblast activation plays a crucial role in the development of pulmonary fibrosis. During fibrogenesis, the lung-resident fibroblast undergoes a dynamic fibroblast-myofibroblast transition (FMT), marked by extracellular matrix secretion and contraction of actomyosin-based stress fibres [6]. Alteration in fibroblast metabolism, particularly glycolysis, has emerged as an important mechanism to ultimately regulate phenotypes. Enhanced glycolysis has been linked to rapid cell growth, proliferation, and ECM secretion of myofibroblasts [7]. Transforming growth factor-β (TGF-β), the principal mediator of FMT, drives glycolytic reprogramming by upregulating key glycolytic enzymes, thereby sustaining heightened glycolysis and promoting fibroblast activation during fibrogenesis [8–12]. Additionally, the abnormal activation of the glycolytic pathway can lead to the accumulation of extracellular lactate and promote pro-fibrotic events [13, 14]. Furthermore, the augmentation of aerobic glycolysis has been shown to facilitate the synthesis and secretion of collagen [15]. Consequently, targeting glycolysis reprogramming to block fibroblast activation has significant implications for therapies aimed at lung fibrosis.

Vitamin D is a fat-soluble secosteroid, which is widely recognized for its vital role in calcium and bone metabolism [16]. The active form of Vitamin D, 1,25(OH)_2_D_3_, interacts with the Vitamin D receptor (VDR) to form a heterodimer with the retinoic X receptor. This complex then binds to Vitamin D response elements in DNA, thereby regulating gene expression in either a positive or negative manner [17]. Additionally, because VDR has been identified in numerous tissues, the non-skeletal actions of Vitamin D have received considerable attention. Several studies have elucidated the significance of Vitamin D in various pulmonary diseases, including asthma, chronic obstructive pulmonary disease (COPD), and pulmonary fibrosis [18–20]. Mechanically, Vitamin D may exhibit anti-fibrotic effects by inhibiting multiple signaling pathways associated with fibrosis, including the TGF-β/Smad signaling pathway [21]. Concurrently, Vitamin D deficiency exacerbates BLM-induced pulmonary fibrosis [22]. Despite the findings above, the exact mechanisms through which Vitamin D affects the onset and progression of pulmonary fibrosis remain insufficiently understood.

In this study, we demonstrated that Vitamin D suppresses TGF-β1-induced fibroblast activation by partly blocking glycolysis. RNA sequencing and subsequent experiments revealed that the expression of TK1 increased after TGF-β1 treatment but decreased in MRC-5 cells and mouse primary lung fibroblasts treated with Vitamin D. Consistent with the male predominance observed in occupational pulmonary fibrosis, male C57BL/6 mice were utilized to enhance clinical relevance [23]. Further in vitro and in vivo data confirmed that TK1 could dramatically enhance PFKFB3 expression at the protein level, thereby contributing to pulmonary fibrosis via regulating fibroblast glycolysis and activation. In conclusion, our findings highlight the substantial therapeutic potential of Vitamin D and its downstream molecules in managing pulmonary fibrosis.

## Materials and methods

### Animal studies

The animal experimental procedures conducted in this study were granted ethical approval by the Institutional Animal Care Committee at Binzhou Medical University (Ethical approval number: 2021336). Given the male predominance observed in human silicosis cases and the existing literature indicating that occupational workers are predominantly young to middle-aged adults, six-week-old male C57BL/6 mice were selected as the experimental model [24]. The mice were obtained from Pengyue Co., Ltd. (Jinan, China) and maintained in a controlled temperature environment with a 12-hour light/dark cycle, with ad libitum access to food and water throughout the study.

To establish experimental models representing distinct stages of fibrotic progression, mice were subjected to varying times of silica exposure. Specifically, silicosis-induced groups received a single intratracheal administration of 50 mg/kg crystalline SiO_2_ (0.5∼ 10 μm; Sigma-Aldrich, USA) particles suspended in 50 µL of sterile saline solution, delivered under standardized pentobarbital anesthesia (Dainippon Sumitomo Pharma, Osaka, Japan). Mice were sacrificed 7, 14, and 28 days after silica exposure, and the lungs were collected and stored at -80 °C.

For the dose-response investigation, mice exposed to silica were randomly allocated to the following groups: control group, SiO₂ group, and five treatment groups receiving SiO₂ in conjunction with Vitamin D at doses of 2.5, 5, 10, 20, and 50 µg/kg. Mice in the Vitamin D-treated groups received intraperitoneal injections of 100 µL of sterile saline containing the corresponding dose of 1,25(OH)₂D₃ every three days. All animals were euthanized on day 28 post-exposure for further analysis.

To examine the role of Vitamin D and 3PO in silica-induced lung fibrosis, a total of 30 mice were randomly divided into five groups (*n* = 6 per group): control, SiO_2_ group, SiO_2_ + VD group, SiO_2_ + 3PO group, and SiO_2_ + VD + 3PO group. 1,25(OH)₂D₃ (MedChemExpress, USA) was initially dissolved in anhydrous ethanol, 3PO (MedChemExpress, USA) was dissolved in dimethyl sulfoxide (DMSO, Beyotime, China), and subsequently diluted with sterile saline to ensure that the final anhydrous ethanol and DMSO concentration did not surpass 10%, thereby reducing potential toxicological effects in murine models [25, 26]. From the next day after silica exposure, mice in the Vitamin D intervention groups received 1,25(OH)₂D₃ 10 µg/kg every three days, and the 3PO treatment groups received the small molecule inhibitor 3PO at a dosage of 20 mg/kg every three days. This administration was executed via intraperitoneal (i.p.) injection, commencing after exposure to silica dust. After four weeks, all mice were sacrificed, and their lungs were harvested and immediately stored at -80 °C for later analysis.

In parallel, thirty mice were randomly assigned to five groups, each comprising six animals: control group, bleomycin (bleo) group, bleo + VD group, bleo + 3PO group, and bleo + VD + 3PO group. Mice in the bleomycin-treated cohorts received a single intratracheal aerosol administration of bleomycin (2.5 mg/kg; MedChemExpress, USA). Commencing one day post-bleomycin instillation, drug treatments were administered via i.p. injection every three days. The interventions included 1,25(OH)₂D₃ (10 µg/kg; MedChemExpress, USA) and 3PO (20 mg/kg; MedChemExpress, USA). On day 21 following bleomycin administration, lung tissues were harvested for histopathological evaluation.

### Histology

The lung tissues were soaked in 4% formalin for 24 h at room temperature, embedded in paraffin, and sectioned to a thickness of 5 μm. The sections underwent hematoxylin and eosin (H&E) staining, Masson trichrome staining, and immunohistochemical examination according to the instructions of the manufacturer. Subsequently, these specimens were analyzed utilizing panoramic scanning electron microscopy. Pulmonary fibrosis was measured quantitatively in sections stained with Masson’s trichrome, using Image J (Fiji) software. The algorithm calculated the ratio of the collagen fiber area (stained blue) to the overall pulmonary tissue area, excluding airspaces.

### Cell culture and treatment

In this study, mouse primary lung fibroblasts were isolated following the completion of animal experiments. Additionally, MRC-5 cells were procured from Procell Co., Ltd. (Wuhan, China). To maintain the cells in an optimal state, distinct culture media were employed for each cell type. The primary mouse lung fibroblasts and MRC-5 cells were cultured in DMEM (Life Technologies/Gibco, Grand Island, NY) and MEM (Life Technologies/Gibco, Grand Island, NY), respectively. Both media were supplemented with 10% fetal bovine serum (Life Technologies/Gibco, Grand Island, NY), 100 U/mL penicillin, and 100 µg/mL streptomycin (Life Technologies/Gibco, Gaithersburg, MD). All cells were maintained at 37 °C in a humidified incubator with 5% CO₂. Fibroblast activation was induced by treating cells with 5 ng/mL recombinant TGF-β1 (Peprotech, USA) for 48 h. To evaluate the potential anti-fibrotic effects of Vitamin D, cells were pretreated with 100 µM 1,25(OH)_2_D_3_ (Sigma-Aldrich, USA) for 24 h prior to the addition of TGF-β1. To assess glycolysis inhibition, MRC-5 cells and mouse primary lung fibroblasts were pretreated with 1, 3, or 10mM 2-Deoxy-d-glucose (2-DG, MedChemExpress, USA) for 1 h before TGF-β1 exposure. Furthermore, the impact of PFKFB3 was studied by treating fibroblasts with 10 µM 3PO (MedChemExpress, USA) for 1 h, followed by treatment with the TK1 plasmid for 96 h.

### Cell transfection

Control siRNA, VDR siRNA, TK1 siRNA, and TK1 plasmid were designed and synthesized by GenePharm (Shanghai, China). Following the manufacturer’s specifications, cells exhibiting 60% confluence were subjected to transfection utilizing small interfering RNA (siRNA) or plasmid, facilitated by the application of riboFECTCP Reagent (RiboBio, Guangzhou, China). The cells were subjected to transfection with VDR-siRNA for a duration of 96 h or were stimulated with TGF-β1 (5 ng/ml) for 48 h, subsequent to a 24-hour transfection period.

### Western blot analysis

Protein extracts were obtained from cultured cell lines using RIPA lysis buffer with a phosphatase inhibitor (Beyotime, China). Total protein from murine tissues was extracted using T-PER Tissue Protein Extraction Reagent (Thermo Fisher Scientific Pierce, USA). Subsequently, the protein concentration was quantified utilizing the BCA Protein Assay kit (Beyotime, Shanghai, China), according to the manufacturer’s protocol. The protein samples were separated via 10% SDS-PAGE (Beyotime, Shanghai, China) and transferred to PVDF membranes (Millipore, USA). After blocking in 5% fat-free milk for 1 h, the membranes were incubated overnight at 4 °C with the following primary antibodies. Following a 15-minute wash with TBST, the membranes were incubated with secondary antibodies for 1 h at room temperature. Subsequently, the membranes underwent a 45-minute washing step with TBST and were imaged immediately utilizing the Tanon Chemiluminescence Instrument (Tanon, Shanghai, China). The antibody information was documented in Supplementary File 2, Table S3.

### qRT-PCR

Total RNAs from lung tissues or cells were extracted using TRIzol reagent (TianGen). Complementary DNA (cDNA) was produced using HiScript^®^ II Q RT SuperMix for qPCR Kit according to the manufacturer’s protocol and used in subsequent real-time qPCR reactions. Real-time PCR was performed with an AceQ^®^ qPCR SYBR^®^ Green Master Mix kit (Vazyme Biotech Co., Ltd., Nanjing, China) to determine relative gene expression. The quantification of transcript levels was achieved through the 2^−ΔΔCt^ method, with normalization performed against GAPDH as an internal control.

### Immunofluorescence staining

MRC-5 cells and mouse primary lung fibroblasts were subjected to fixation using 4% paraformaldehyde for 30 min, followed by three washes with PBS. Subsequently, the cells were blocked with goat serum for one hour and incubated overnight at 4 °C with primary antibodies targeting alpha-smooth muscle actin (α-SMA, ab124964, Abcam), Collagen I (ab34710, Abcam; 67288-1-Ig, proteintech, China), or TK1 (A5612, ABclonal, China). Following the washing of cells with PBST, they were labeled in the dark for 1 h with either Cy3-conjugated goat anti-rabbit, FITC-conjugated goat anti-rabbit, or FITC-conjugated goat anti-mouse secondary antibodies (Beyotime, China). Subsequently, the nuclei were stained with DAPI. Imaging was performed using a Nikon fluorescent microscope.

### Cell proliferation assay

MTT Cell Proliferation and Cytotoxicity Assay Kit (Beyotime, China) was evaluated utilizing cell viability, strictly following the manufacturer’s instructions. Following treatment, the cells were treated with 10 µL of a 5 mg/mL MTT solution and incubated at 37 °C for 4 h. After removing the supernatants, the resultant MTT formazan precipitate was solubilized in 100 µL of a designated dissolving solution at 37 °C for an additional 4 h. The absorbance of each well was then measured at 570 nm using a microplate reader (BioTek, Winooski, VT, USA).

Cell proliferation was quantitatively evaluated using the EdU Cell Proliferation Kit (Beyotime, China). An EdU working solution was formulated by mixing the cell culture medium with the EdU reagent at a precise ratio of 2500:1. Subsequently, this solution was added to the Petri dishes and incubated at 37 °C for 2 h. After incubation, the cells were subjected to fixation, permeabilization, and staining procedures. Fluorescence imaging was performed using a Nikon microscope to visualize cellular proliferation in detail.

### Collagen gel contraction assay

Collagen gels were synthesized in 24-well plates using a rat tail-derived type I collagen solution (Xinyou Biotechnology Co., Ltd., Hangzhou, China; Cat#200110) at a 1–2 mg/mL concentration range. The collagen solution was supplemented with 0.1 M NaOH and 10× PBS. Following polymerization, MRC-5 fibroblasts were seeded into the gels and incubated at 37 °C in a 5% CO_2_ atmosphere for 48 h. The outcomes were quantitatively assessed based on changes in the gel surface area.

### Metabolic studies

Following the manufacturer’s guidelines, metabolic studies were conducted utilizing specific assay kits. Glucose consumption was quantitatively measured employing a glucose assay kit (BioVision, Milpitas, CA, USA). The lactate production was determined by applying a Lactic Acid assay kit (Jiancheng, China, A019-2-1). ATP production was quantified utilizing an ATP assay kit (Beyotime, China, S0026). Additionally, collagen content in lung tissues was assessed using a Hydroxyproline content assay kit (Jiancheng, China, A030-2-1).

Extracellular acidification rate (ECAR) assays (Elabscience, E-BC-F069) were performed using a microplate reader (BioTek, Winooski, VT, USA). MRC-5 cells were seeded on a microplate (2 × 10^4^ cells/well). After inoculation, the cells were put into a 37℃ carbon dioxide incubator overnight. A volume of 100µL of the working solution was added to each well. Fluorescence measurements were conducted using a fluorescence enzyme marker, with excitation and emission wavelengths set at 490 nm and 535 nm, respectively. The fluorescence intensity was recorded at 2-minute intervals over a 120-minute duration.

### RNA-seq profiling

MRC-5 cells were subjected to total RNA extraction utilizing TRIzol reagent (TianGen). An input of 1 µg of RNA per sample was employed for subsequent RNA sample preparations. Ruixing Biotechnology Co., Ltd. (Wuhan, China) performed the creation and sequencing of the RNA libraries. Cells were categorized into three experimental groups, each comprising four biological replicates: control, TGF-β1, and TGF-β1 + VD. The integrity and purity of RNA were checked prior to library preparations. A NEBNext^®^ UltraTM RNA Library Prep Kit for Illumina was used according to the manufacturer’s recommendations to create the sequencing libraries. Then, total RNA from lung fibroblasts was sequenced on an [Illumina NovaSeq 6000] platform to generate 150 bp paired-end reads. The high-quality trimmed reads were aligned to the GRCh38 (human) reference genome and transcriptome using HISAT2 software. Differential expression analysis was conducted using the DESeq2 R package (version 1.6.3), with differentially expressed genes (DEGs) defined as those with an adjusted fold change (FC) ≥ 2 or ≤ 0.5 and false discovery rate (FDR) < 0.05. Gene ontology (GO) enrichment analysis for differentially expressed genes (DEGs) was conducted using the DAVID Bioinformatics Resources (version 6.8). The results were visualized with the GOplot R package (version 1.0.2). The analysis included three categories of GO terms: biological process (BP), cellular component (CC), and molecular function (MF). The top BP terms were statistically assessed with a significance level of *P* < 0.05 and a fold change (FC) of ≤ − 1.5 or ≥ 1.5.

### Enzyme-linked immunosorbent assay (ELISA) assays

The concentrations of IL-1β, IL-6, and TNF-α in bronchoalveolar lavage fluid (BALF) and pulmonary tissue were quantified. Enzyme-linked immunosorbent assay (ELISA) kits (ELK Biotechnology) were employed in accordance with the manufacturer’s protocols.

### Statistical analysis

Data are expressed as means ± SD. All experiments were conducted at least three times to ensure reliability. The normality of data distribution was evaluated for each experiment, utilising the Shapiro-Wilk test. Parametric tests were employed when data conformed to a normal distribution, whereas non-parametric tests were applied in cases where the data deviated from normality. A two-tailed unpaired Student’s t-test was employed for the comparison between two groups. Significant differences among groups were evaluated using ANOVA followed by Bonferroni correction, employing GraphPad Prism 6.0 software for statistical analysis. A p-value of less than 0.05 was considered to indicate statistical significance.

## Results

### Enhanced glycolysis contributes to pulmonary fibroblast activation

**Fig. 1:**
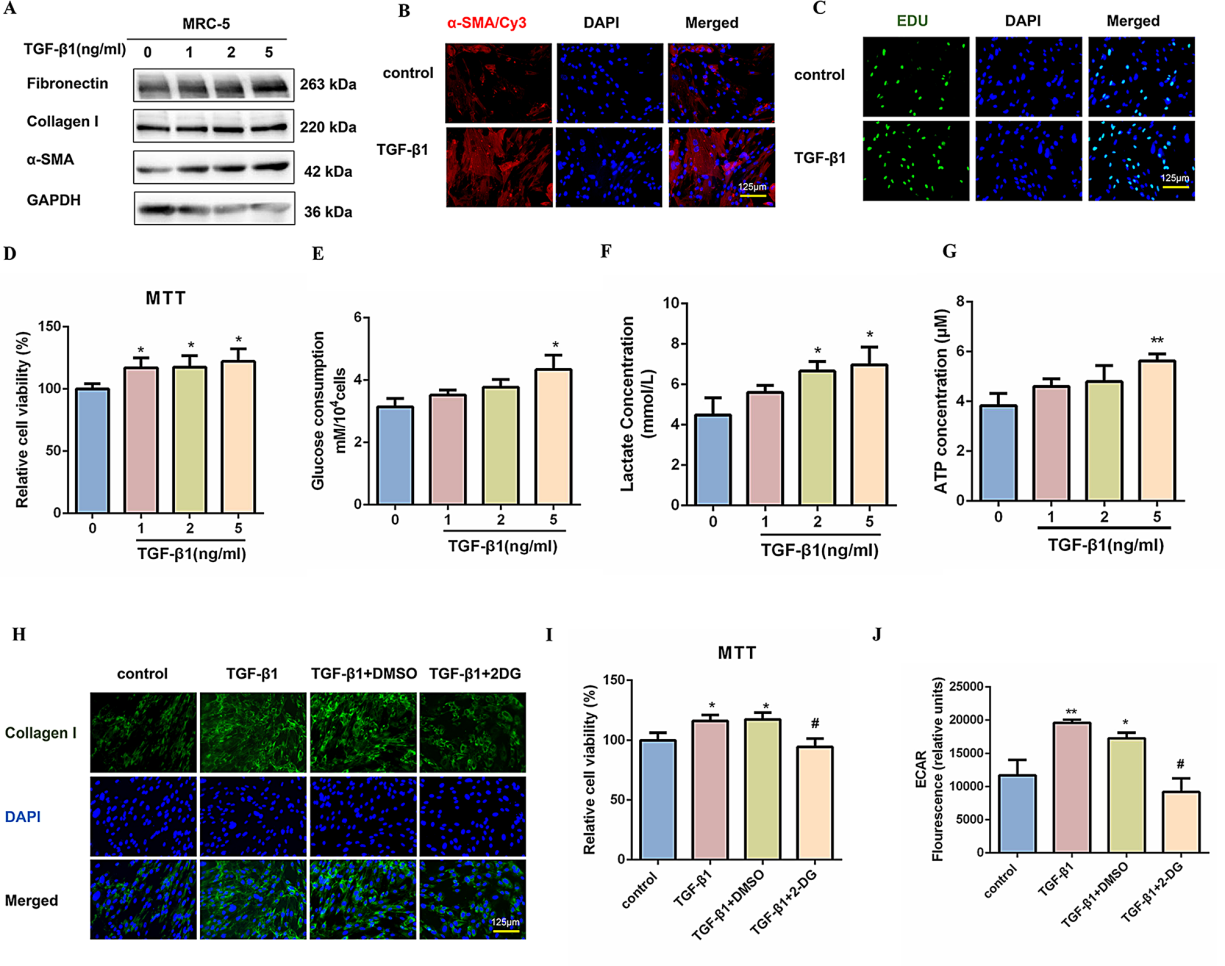
Enhanced glycolysis contributes to pulmonary fibroblast activation. (**A**) Protein level detection of Fibronectin, Collagen I, and α-SMA levels in MRC-5 cells treated with 0, 1, 2, and 5ng/mL of TGF-β1 for 48 h. GAPDH was used as a loading control. The experiments were repeated three times and the results were similar. (**B**) Representative immunofluorescence staining of α-SMA (red) and DAPI (blue) was performed on MRC-5 cells treated with 5 ng/mL of TGF-β1 for 48 h. Scale bar = 125 μm. (**C**) DNA synthesis was assessed using the EdU assay in MRC-5 cells for the control and TGF-β1 (5 ng/mL) treatment groups. Green, EdU; blue, nuclei. Scale bar = 125 μm. (**D**) MRC-5 cells were stimulated with TGF-β1 for 48 h, and cell viability was determined by MTT assay (*n* = 3), *P < 0.05. (**E**-**G**) Glucose consumption, lactate levels, and ATP concentration were measured (*n* = 3), with *P < 0.05 and **P < 0.01 vs. control. (**H**) MRC-5 cells were pretreated with 2-DG at 10 mM for 1 h, then exposed to TGF-β1 for 48 h. Graphic presentation depicting the immunostaining for Collagen I (green) in MRC-5 cells. The nucleus was stained with DAPI. Scale bar = 125 μm. (**I**-**J**) MTT assays were employed to evaluate cell viability, while the ECAR was utilized to assess glycolytic activity (*n* = 3), *P < 0.05, **P < 0.01 vs. the control group, #*P* < 0.05 vs. TGF-β1 + DMSO group. For **D**, **E**, **F**, **G**, **I**, and **J**, one-way ANOVA was used with the Bonferroni multiple comparisons test. Data are presented as mean ± SD. Source data are provided as a Source data file.

Given the pivotal role of fibroblasts in the pathogenesis of pulmonary fibrosis, we established a fibroblast activation cell model utilizing TGF-β1 stimulation. MRC-5 cells were treated with varying concentrations of TGF-β1 (0, 1, 2, and 5 ng/mL) over 48 h to induce fibroblast activation in vitro. As anticipated, western blot analyses demonstrated a dose-dependent increase in profibrotic factors, specifically Fibronectin, Collagen I, and α-SMA (Fig. 1A and S1A). Furthermore, treatment with 5 ng/mL TGF-β1 achieved a saturation level of these profibrotic markers. Immunofluorescence staining with α-SMA antibodies showed a significant elevation in α-SMA-positive fibroblasts after TGF-β1 treatment, along with enhanced proliferative activity (Fig. 1B-D and S1B). Additionally, the immunofluorescence analysis of mouse primary lung fibroblasts confirmed the increased expression levels of Collagen I and α-SMA following treatment with TGF-β1 (Fig. S1C, D). Consequently, in subsequent experiments, the cells were treated with 5 ng/mL TGF-β1 for 48 h.

Additionally, extracellular glucose utilization, lactate production, and ATP levels in MRC-5 cells and mouse primary lung fibroblasts exposed to TGF-β1 treatment were significantly elevated (Fig. 1E-G and S1E-G). Further analysis elucidated that pharmacological suppression of glycolysis via 2-DG attenuated TGF-β1-driven fibrogenic responses, including the synthesis of fibrosis markers and fibroblast hyperproliferation (Fig. 1H, I and S1H, I). Furthermore, we determined ECAR as a measure of glycolytic rate. In alignment with our hypotheses, the application of 2-DG reduced the elevated ECAR observed in TGF-β1-treated cells (Fig. 1J). This observation was also substantiated by decreased extracellular lactate production and ATP levels conducted on mouse primary lung fibroblasts (Fig. S1J, K). These results collectively indicate that enhanced glycolysis contributes to pulmonary fibroblast activation.

### Vitamin D exerts anti-fibrotic effects by reducing glycolysis

**Fig. 2:**
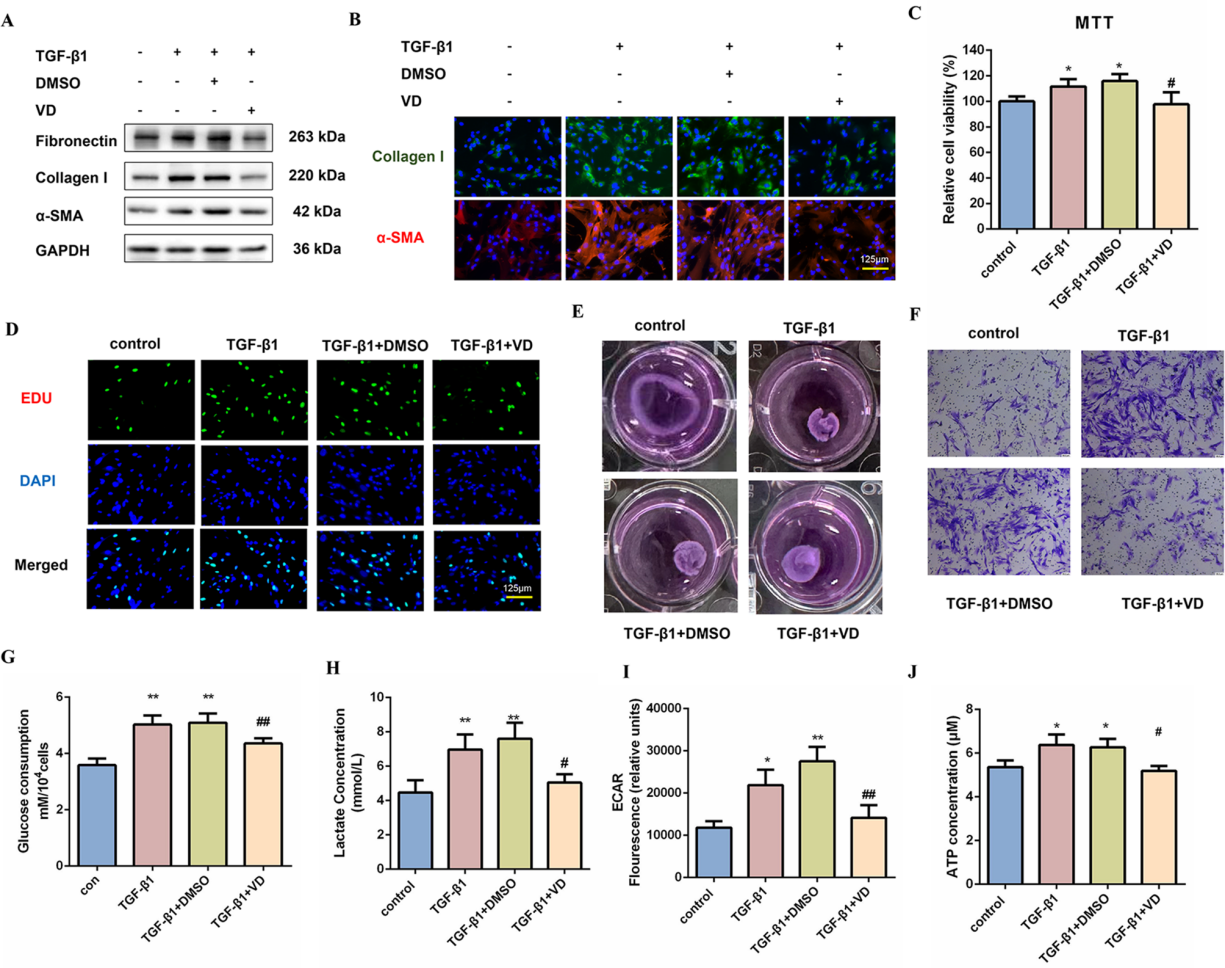
Vitamin D exerts anti-fibrotic effects by reducing glycolysis. (**A**) Western blot analysis was used to examine the expression of Fibronectin, Collagen I, and α-SMA in MRC-5 cells treated with Vitamin D at 100 nM for 24 h before TGF-β1 treatment for 48 h. GAPDH was used as a loading control. The experiments were repeated three times and the results were similar. (**B**) Immunofluorescence staining of Collagen I (green) and α-SMA (red) in MRC-5 cells for the indicated groups. Blue represents nuclear DNA staining by DAPI. Scale bar = 125 μm. (**C**-**D**) The MTT and EdU assays were conducted on MRC-5 cells treated with Vitamin D at 100 nM for 24 h before TGF-β1 treatment for 48 h to assess cell proliferative ability (*n* = 3), *P < 0.05 vs. the control group, and #*P* < 0.05 vs. TGF-β1 + DMSO group. Scale bar = 125 μm. (**E**) Contraction assay of the collagen gel complex in the presence or absence of Vitamin D. (**F**) Evaluating cell migration in TGF-β1-treated MRC-5 cells with and without co-incubation of Vitamin D, using the Transwell migration assay. (**G**-**J**) Glucose consumption, lactate concentration, ECAR, and ATP concentration were detected in MRC-5 cells for the indicated groups (*n* = 3), *P < 0.05, **P < 0.01 vs. the control group, #*P* < 0.05, and ##*P* < 0.01 vs. TGF-β1 + DMSO group. For **C**, **G**, **H**, **I**, and **J**, a one-way ANOVA was used, followed by the Bonferroni multiple comparisons test. Data are presented as mean ± SD. Source data are provided as a Source data file.

To clarify the role of Vitamin D in the progression of fibroblast activation, MRC-5 cells and mouse primary lung fibroblasts were treated with 1,25(OH)_2_D_3_ at a concentration of 100nM for 24 h before TGF-β1 treatment for 48 h. Western blot analysis demonstrated that treatment with Vitamin D significantly mitigated the upregulation of Fibronectin, Collagen I, and α-SMA expression levels subsequent to TGF-β1 treatment (Fig. 2A and S2A, B). Consistent with the results mentioned above, immunofluorescence staining revealed that Vitamin D inhibited the expression of Collagen I and α-SMA (Fig. 2B and S2C, D). Moreover, Vitamin D significantly attenuated TGF-β1-induced fibroblast proliferation, as confirmed by both MTT viability assays and EdU incorporation analysis (Fig. 2C, D). To further verify the anti-fibrotic effects of Vitamin D in TGF-β1-treated MRC-5 cells, we evaluated fibrotic-related functions, specifically focusing on contractile capacity and cellular migration. Collagen gel contraction assay and transwell results showed that TGF-β1 treatment significantly enhanced collagen gel contraction and stimulated cell migration, whereas Vitamin D significantly attenuated this effect (Fig. 2E, F). Interestingly, our findings indicate that the glycolysis stimulated by TGF-β1 was inhibited in MRC-5 cells and mouse primary lung fibroblasts following Vitamin D treatment. Metabolic analyses revealed a reduction in extracellular glucose utilization, lactate production, extracellular acidification rate, and ATP levels in MRC-5 cells and mouse primary lung fibroblasts treated with Vitamin D (Fig. 2G-J and S2E-G). These results suggest that Vitamin D exerts anti-fibrotic effects by reducing glycolysis.

### TK1 is a downstream target of Vitamin D in lung fibroblasts

**Fig. 3:**
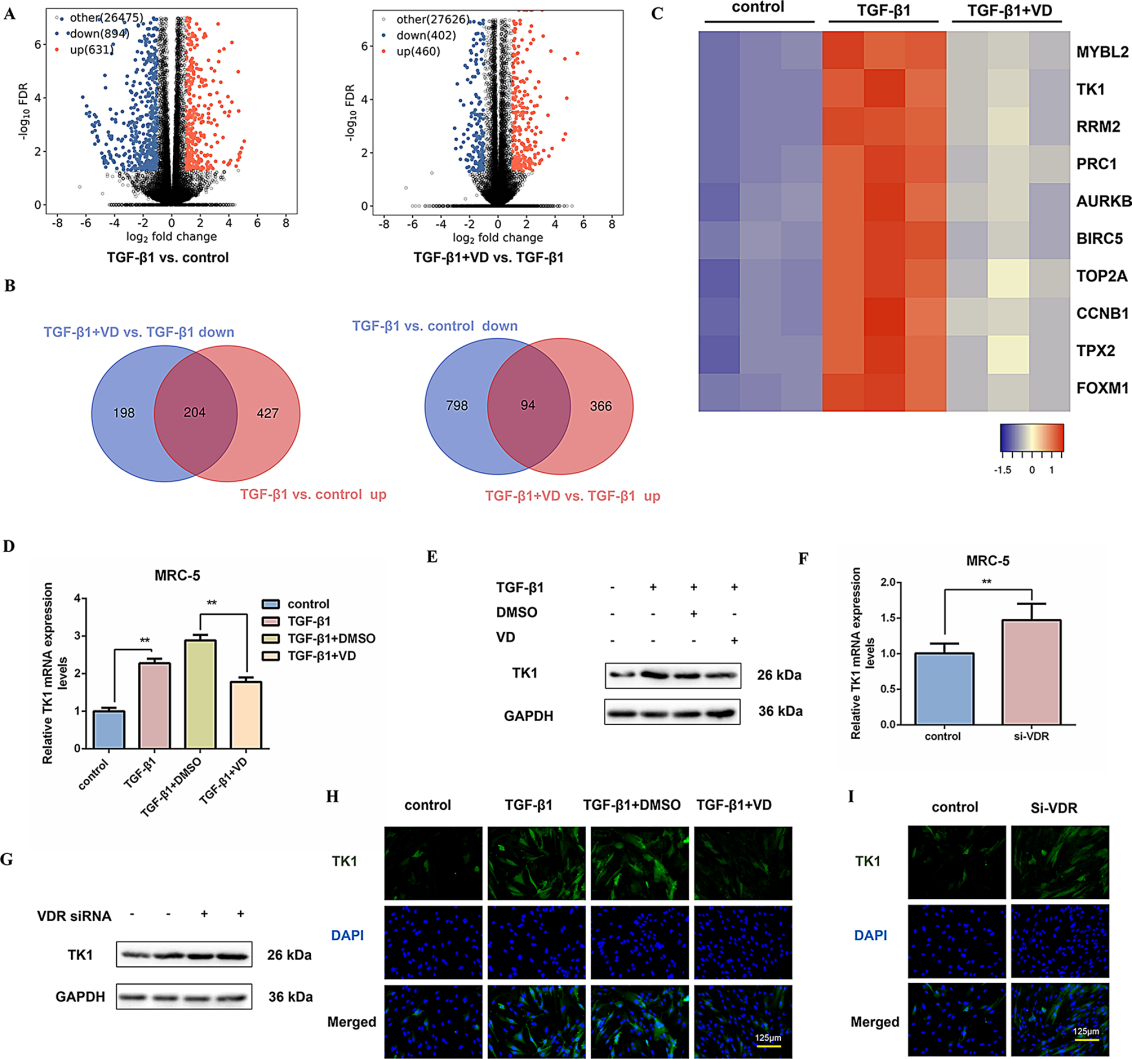
TK1 is a downstream target of Vitamin D in lung fibroblasts. (**A**) The volcano plot illustrates the differential expression of protein-coding genes in MRC-5 cells, with blue dots denoting genes that are significantly downregulated and red dots indicating genes that are significantly upregulated. (**B**) Differentially expressed genes (DEGs) in fibroblasts treated with TGF-β1 versus control and those with Vitamin D co-treatment, with criteria of fold change > 2 and FDR < 0.05. (**C**) Heatmap showing dysregulated genes (red, upregulated genes; blue, downregulated genes) identified by DEGs. (**D**-**E**) TK1 expression was determined by qRT-PCR and Western blot in MRC-5 cells (n = 3), **P < 0.01. GAPDH was used as a loading control. The experiments were repeated three times and the results were similar. (**F**-**G**) Western blot analysis and qRT-PCR were used to quantify the expression of TK1 in MRC-5 cells treated with VDR siRNA for 96 h (*n* = 3), **P < 0.01. GAPDH was used as a loading control. The experiments were repeated three times and the results were similar. (**H**-**I**) Immunofluorescence staining of TK1 was conducted in MRC-5 cells categorized into specified treatment groups. MRC-5 cells were exposed to either Vitamin D at 100 nM for 24 h prior to TGF-β1 treatment for 48 h or VDR siRNA for 96 h. Green represents TK1 staining; blue represents nuclear DNA staining by DAPI. Scale bar = 125 μm. For D, one-way ANOVA was used with the Bonferroni multiple comparisons test. For **F**, a two-tailed t-test was used. Data are presented as mean ± SD. Source data are provided as a Source data file.

To further investigate the downstream mechanism of Vitamin D on TGF-β1-driven fibroblast activation and glycolytic reprogramming, we performed RNA-Seq-based transcriptomic profiling, revealing that TGF-β1 stimulation significantly upregulated 631 genes and downregulated 894 genes. Following co-treatment with Vitamin D, a total of 862 genes were identified as significantly altered, with 402 exhibiting downregulation and 460 exhibiting upregulation (Fig. 3A). Subsequently, we conducted screening to identify genes exhibiting dysregulation. Venn diagram analysis demonstrated that Vitamin D supplementation counteracted the upregulation of 204 genes by TGF-β1. Furthermore, 94 genes were identified at the intersection of those downregulated in the TGF-β1 versus control comparison and those upregulated in the TGF-β1 + VD versus TGF-β1 comparison (Fig. 3B). The heatmap analysis of transcriptional profiles confirmed that TK1, a key regulator of nucleotide metabolism, was transcriptionally activated by TGF-β1 but suppressed upon Vitamin D intervention, indicating that it may be a downstream target of Vitamin D (Fig. 3C). This is further supported by the inhibitory effects of Vitamin D on the expression levels of both TK1 protein and mRNA in MRC-5 cells (Fig. 3D, E and S3A). Furthermore, the successful activation of the VDR signaling pathway was corroborated by a dose-dependent induction of CYP24A1 subsequent to Vitamin D administration, thereby substantiating the functional involvement of the VDR pathway in this process (Fig. S3B).

To reveal the biological roles of TK1 within the context of pulmonary fibrosis, we utilized siRNA specifically targeting the VDR to attenuate its expression in MRC-5 cells for 96 h. Subsequent analyses indicated that the silencing of VDR significantly resulted in an upregulation of both TK1 mRNA and protein levels (Fig. 3F, G and S3C). Additionally, immunofluorescence staining corroborated that Vitamin D treatment inhibited TK1 expression. In contrast, the knockdown of VDR resulted in a marked elevation of TK1 signal intensity (Fig. 3H, I and S3D, E). Collectively, these findings suggest that TK1 is a downstream target of Vitamin D, with its expression negatively regulated by Vitamin D.

### TK1 is involved in fibroblast activation and glycolysis

**Fig. 4:**
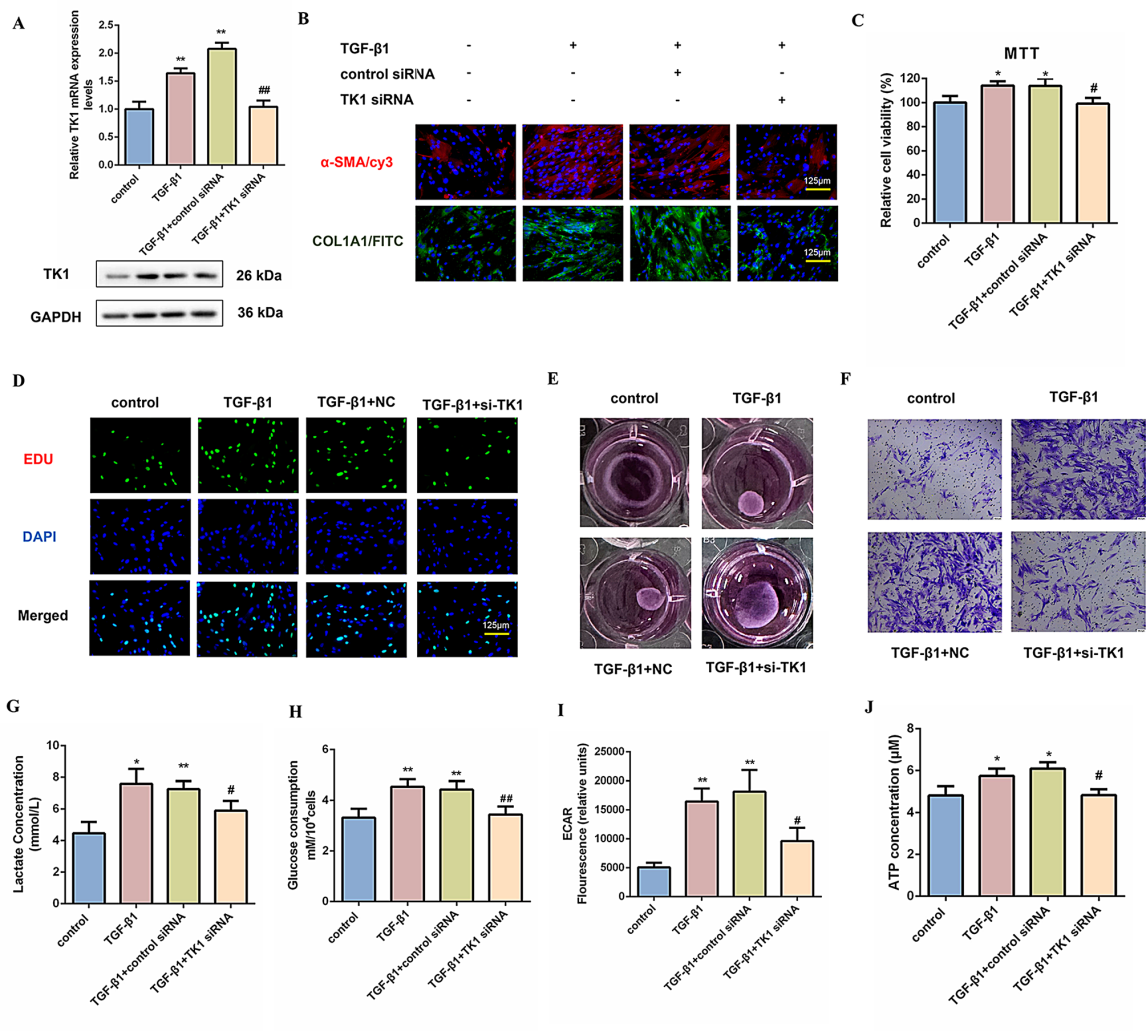
TK1 is involved in fibroblast activation and glycolysis. (**A**) Western blot and qRT-PCR analysis of TK1 in MRC-5 cells transfected with TK1 siRNA for 24 h, then treated with 5ng/mL TGF-β1 for 48 h (*n* = 3), with **P < 0.01 vs. the control group, and ##*P* < 0.01 vs. TGF-β1 + control siRNA group. GAPDH was used as a loading control. The experiments were repeated three times and the results were similar. (**B**) The expression of α-SMA (red) and Collagen I (green) was assessed via immunofluorescence staining to show fibroblast activation. Scale bar = 125 μm. (**C**-**D**) MTT and EDU assays evaluated the proliferative capacity of MRC-5 cells transfected with TK1 siRNA for 24 h, followed by treatment with 5 ng/mL TGF-β1 for 48 h (*n* = 3). The results showed *P < 0.05 vs. the control group and #*P* < 0.05 vs. the TGF-β1 + control siRNA group. Scale bar = 125 μm. (**E**) MRC-5 cell contraction was measured using the collagen gel assay. (**F**) Cell migration ability in TGF-β1-treated MRC-5 cells with or without TK1 siRNA co-incubation using the Transwell migration assay. (**G**-**J**) Lactate concentration, glucose consumption, ECAR, and ATP concentration were assessed in MRC-5 cells across the experimental groups (*n* = 3), with *P < 0.05, **P < 0.01 vs. the control group, and #*P* < 0.05, ##*P* < 0.01 vs. TGF-β1 + control siRNA group. For **A**, **C**, **G**, **H**, **I**, and **J**, one-way ANOVA was used with the Bonferroni multiple comparisons test. Data are presented as mean ± SD. Source data are provided as a Source data file.

To investigate the functional role of TK1 in fibroblast activation, we silenced TK1 expression through siRNA transfection in MRC-5 cells and mouse primary lung fibroblasts. Stimulation with TGF-β1 at a concentration of 5 ng/mL for 48 h induced significant transcriptional activation of TK1, whereas combined treatment with TGF-β1 and TK1 siRNA significantly reduced this induction, as evidenced by qRT-PCR and Western blot (Fig. 4A and S4A). TK1 knockdown concomitantly suppressed TGF-β1-driven expression of activated fibroblast markers, α-SMA and Collagen I (Fig. 4B and S4B-D). Moreover, proliferation assays revealed that the reduction of TK1 expression significantly decreased the proliferation activity of MRC-5 cells induced by TGF-β1 (Fig. 4C, D). Functional analyses further demonstrated that TK1 depletion diminished TGF-β1-enhanced contractile and migratory capacity (Fig. 4E, F). Interestingly, our findings indicate that the glycolysis process stimulated by TGF-β1 was significantly inhibited in MRC-5 cells and mouse primary lung fibroblasts after the knockdown of TK1. Lactate production, glucose consumption, ECAR, and ATP levels were considerably reduced (Fig. 4G-J and S4E-G). These results suggest that TK1 is involved in fibroblast activation and glycolysis.

### PFKFB3 mediates the function of TK1 to regulate fibroblast activation and glycolytic reprogramming

**Fig. 5:**
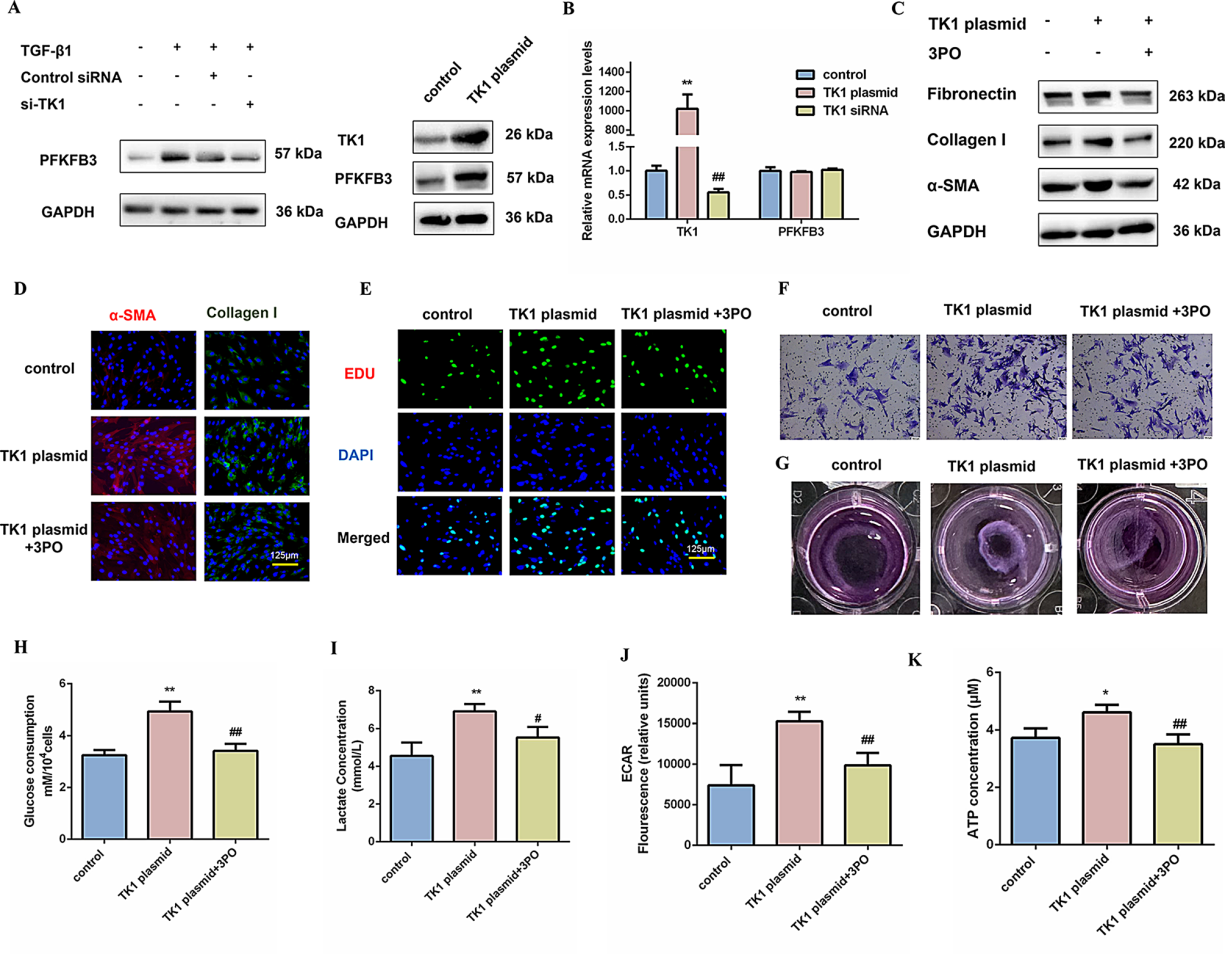
PFKFB3 mediates the function of TK1 to regulate fibroblast activation and glycolytic reprogramming. (**A**-**B**) Western blot and qRT-PCR analysis of the protein expression and mRNA levels of TK1 and PFKFB3 in treated MRC-5 cells for the indicated groups of TK1 siRNA for 24 h, followed by treatment with 5 ng/mL TGF-β1 for 48 h or TK1 plasmid for 96 h (*n* = 3), with **P < 0.01 vs. the control group, and ##*P* < 0.01 vs. TK1 plasmid group. GAPDH was used as a loading control. The experiments were repeated three times and the results were similar. (**C**) Western blot detected Fibronectin, Collagen I, and α-SMA levels in MRC-5 cells treated with 3PO (10 µM) for 1 h, followed by TK1 plasmid treatment for 96 h. GAPDH was used as a loading control. The experiments were repeated three times and the results were similar. (**D**) Immunofluorescence staining of α-SMA and Collagen I in MRC-5 cells for the indicated groups. Red represents α-SMA staining; green represents Collagen I staining; blue represents nuclear DNA staining by DAPI. Scale bar = 125 μm. (**E**-**G**) Fibroblast proliferation, migration, and contraction were determined. Scale bar = 125 μm. (**H**-**K**) Glucose consumption, lactate concentration, glycolytic rate, and ATP concentration were quantitatively assessed in MRC-5 cells across the specified experimental groups (*n* = 3), *P < 0.05, **P < 0.01 vs. the control group, and #*P* < 0.05, ##*P* < 0.01 vs. TK1 plasmid group. For B, a 2-way ANOVA was used. For **H**, **I**, **J**, and **K**, one-way ANOVA was used with the Bonferroni multiple comparisons test. Data are presented as mean ± SD. Source data are provided as a Source data file.

To elucidate the regulatory role of TK1 in fibroblast activation and metabolic reprogramming, we examined its interaction with the glycolytic regulator PFKFB3. The combination of TGF-β1 with TK1 siRNA transfection significantly suppressed PFKFB3 protein expression in MRC-5 fibroblasts, while transfection with a TK1 plasmid led to an upregulation of this protein level (Fig. 5A and S5A). This coordinated regulation designates PFKFB3 as a downstream metabolic effector of TK1 signaling, establishing a hierarchical regulatory relationship. In contrast, the mRNA expression levels of PFKFB3 remained constant irrespective of the modulation of TK1 levels, whether upregulated or downregulated (Fig. 5B). This observation suggests that TK1 exerts a regulatory influence on the post-transcriptional regulation of PFKFB3.

To further elucidate the biological implications of increased glycolysis, we employed a well-characterized PFKFB3 inhibitor, 3PO, to diminish glycolytic activity. As expected, overexpression of TK1 increased the expression of fibrotic markers, including Fibronectin, Collagen I, and α-SMA, effects that were reversed by the PFKFB3 inhibitor 3PO, further confirming PFKFB3 as a downstream mediator (Fig. 5C, D and S5B-E). Meanwhile, treatment with 3PO effectively mitigated the cell proliferation, migration, and contractile capacity prompted by the overexpression of TK1 (Fig. 5E-G). Metabolic flux analysis revealed attenuated TK1-driven glycolytic flux upon PFKFB3 inhibition, evidenced by reduced glucose consumption, lactate accumulation, ECAR, and ATP production (Fig. 5H-K and S5F-H). These data established that PFKFB3 mediates the function of TK1 to regulate fibroblast activation and glycolytic reprogramming.

### Silica-induced pulmonary fibrosis is linked to Vitamin D and glycolytic dysregulation

A murine model of silica-induced pulmonary fibrosis was established via a single intratracheal instillation of silica suspension (50 mg/kg in sterile saline) to further verify the roles of TK1 and PFKFB3 in vivo. H&E staining demonstrated time-dependent alveolar disruption and inflammatory infiltration, culminating in consolidated fibrosis (Fig. S6A). In addition, Masson’s trichrome staining analysis highlighted marked peribronchial and interstitial collagen deposition by day 28 (Fig. S6B and S6L), further validated by elevated hydroxyproline content (Fig. S6C). Western blot also demonstrated the upregulated expression of fibrotic markers, including Fibronectin, Collagen I, and α-SMA in mice subjected to silica (Fig. S6D and S6M). This signifies the successful establishment of a pulmonary fibrosis model induced by silica exposure.

Subsequently, we noted a significant down-regulation of serum 25-(OH)-D_3_ in the 28-day group (Fig. S6E). Immunohistochemistry identified upregulation of TK1 in fibrotic foci (Fig. S6F). Moreover, lactate levels were also found to be elevated after silica exposure (Fig. S6G). Given the pivotal role of fibroblasts in the pathogenesis of pulmonary fibrosis, primary lung fibroblasts derived from mice were isolated and cultured for subsequent experimental investigations. In primary lung fibroblasts, fibrogenic markers demonstrated persistent upregulation during activation, alongside enhanced expression of TK1 and PFKFB3 (Fig. S6H, I and S6N). Meanwhile, metabolic assays confirmed enhanced glucose consumption and lactate production (Fig. S6J, K). These findings collectively implicate that silica-induced pulmonary fibrosis is linked to Vitamin D and glycolytic dysregulation.

To evaluate the therapeutic potential of Vitamin D in silica-induced pulmonary fibrosis, a dose-response study was conducted utilizing a murine model. Following intratracheal instillation of SiO₂, the mice were administered Vitamin D at varying dosages (2.5, 5, 10, 20, and 50 µg/kg) at three-day intervals over four weeks (Fig. S6O). All treated groups maintained body weights within normal physiological limits and exhibited no signs of toxicity, indicating good tolerability of the intervention (Fig. S6P). H&E and Masson’s trichrome staining demonstrated that Vitamin D administration alleviated the pathological alterations caused by silica exposure (Fig. S6Q, R). Specifically, the treatment exhibited a dose-dependent reduction in alveolar structural damage, infiltration of inflammatory cells, and collagen deposition. Notably, the most significant diminution in fibrotic pathology was observed at a dosage of 10 µg/kg. Consistent with these observations, enzyme-linked immunosorbent assay (ELISA) measurements of bronchoalveolar lavage fluid (BALF) and lung tissue indicated that silica exposure led to a substantial increase in pro-inflammatory cytokines, including interleukin-6 (IL-6), interleukin-1β (IL-1β), and tumor necrosis factor-alpha (TNF-α), relative to controls (Fig. S6S-W). Vitamin D treatment significantly mitigated these cytokine elevations, with the most pronounced effects observed at dosages of 10 and 20 µg/kg. Taken together, these results suggest that Vitamin D alleviates silica-induced pulmonary inflammation and fibrosis. Based on these findings, a dosage of 10 µg/kg was selected for subsequent experimental validation.

### Combined Vitamin D and 3PO alleviates silica-induced pulmonary fibrosis in vivo

**Fig. 6:**
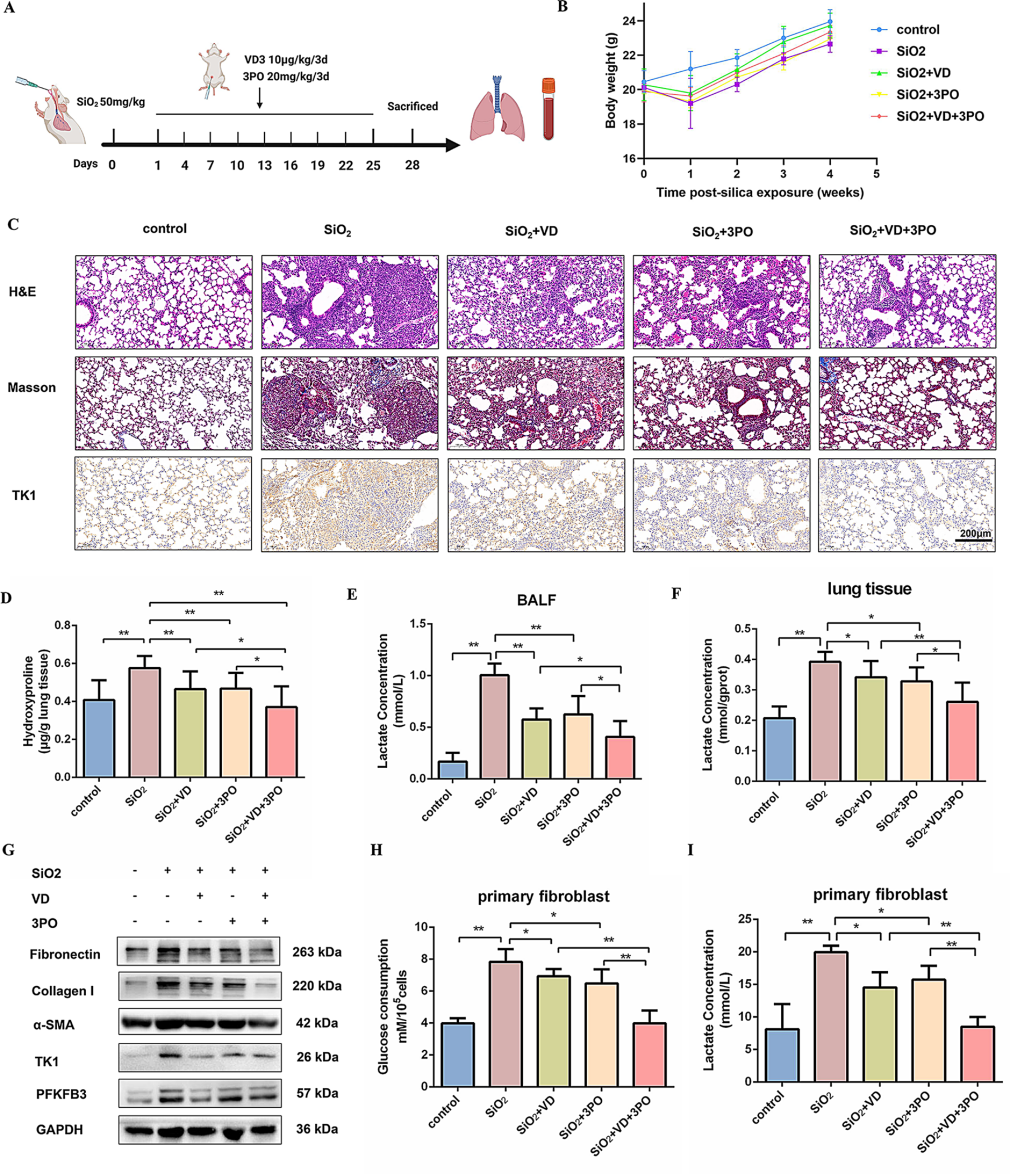
Combined Vitamin D and 3PO alleviates silica-induced pulmonary fibrosis in vivo. (**A**) Strategy for administering Vitamin D, 3PO, or a synergistic combination in a silica-induced pulmonary fibrosis murine model. (**B**) Body weight (g), mean ± SEM. (**C**) H&E staining, Masson staining, and immunohistochemical staining of TK1. (**D**) The quantification of hydroxyproline content was assessed in lung tissues (*n* = 4), *P < 0.05, **P < 0.01. (**E**-**F**) Lactate concentrations were detected in the alveolar lavage fluid of mice and the lung tissue in the indicated groups (*n* = 4), *P < 0.05, **P < 0.01. (**G**) Fibronectin, Collagen I, α-SMA, TK1, and PFKFB3 expression levels were detected by western blot analysis. GAPDH was used as a loading control. The experiments were repeated three times and the results were similar. (**H**-**I**) Glucose consumption and lactate levels were measured in primary fibroblast cultures that received Vitamin D or 3PO (*n* = 4), *P < 0.05, **P < 0.01. For **B**, a 2-way repeated measures ANOVA was used. For **D**, **E**, **F**, **H**, and **I**, one-way ANOVA was used with the Bonferroni multiple comparisons test. Data are presented as mean ± SD. Source data are provided as a Source data file.

In a murine model of silica-induced pulmonary fibrosis, the roles of Vitamin D and 3PO in the progression of pulmonary fibrosis were evaluated through the administration of Vitamin D, the PFKFB3 inhibitor 3PO, or their combination. Starting from the initiation of silica dust treatment, mice in the intervention group received intraperitoneal injections of Vitamin D (10 µg/kg/3d), 3PO (20 mg/kg/3d), or a combination of Vitamin D and 3PO for 28 days (Fig. 6A). Compared to silica-exposed controls, Vitamin D and 3PO therapy demonstrated mitigation of body weight loss (Fig. 6B). The H&E staining of lung sections, the Masson trichrome staining of interstitial collagen, and the immunohistochemical assays of TK1 collectively confirmed that Vitamin D and 3PO significantly reduced silica-induced lung fibrosis (Fig. 6C and S7A). Additionally, the level of lung hydroxyproline showed a significant reduction, most markedly within the combination therapy group (Fig. 6D). The same phenomenon was noted for lactate content in alveolar lavage fluid and lung tissue (Fig. 6E, F). Furthermore, Western blot analysis revealed that Vitamin D, either administered independently or in conjunction with 3PO, markedly decreased the expression of fibrosis markers, including Fibronectin, Collagen I, and α-SMA, as well as TK1 and PFKFB3 (Fig. 6G and S7B). The treatment with Vitamin D and 3PO resulted in a significant reduction in glucose consumption and lactate levels, as evidenced in primary fibroblast cultures (Fig. 6H, I).

Meanwhile, the results of the ELISA assays revealed a significant decrease in the levels of pro-inflammatory cytokines, specifically IL-1β, IL-6, and TNF-α, in BALF and pulmonary tissue specimens subsequent to the administration of Vitamin D and 3PO (Fig. S7C-G). Furthermore, exposure to silica was found to markedly upregulate the mRNA expression of matrix metalloproteinases MMP2 and MMP8, while MMP7 was downregulated in fibrotic lung tissues compared to controls. Significantly, Vitamin D treatment effectively reversed these alterations, bringing their expression levels closer to those observed in normal conditions (Fig. S7H-J). These results support that combined Vitamin D and 3PO treatment attenuates silica-induced fibrosis by modulating glycolytic pathways.

### Combined Vitamin D and 3PO alleviates bleomycin-induced pulmonary fibrosis in vivo

**Fig. 7:**
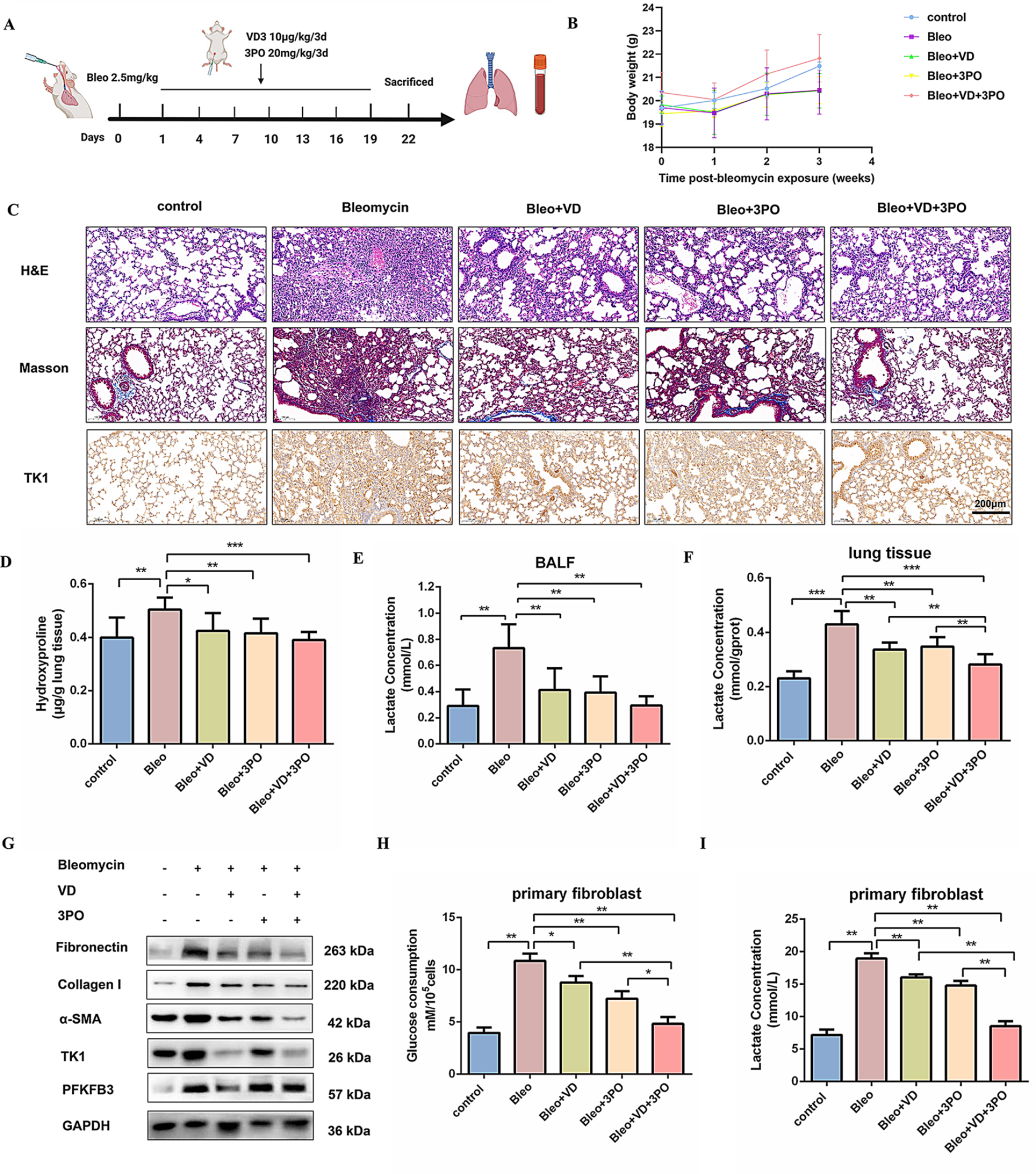
Combined Vitamin D and 3PO alleviates bleomycin-induced pulmonary fibrosis in vivo. (**A**) Strategy for administering Vitamin D, 3PO, or a synergistic combination in a bleomycin-induced pulmonary fibrosis mouse model. (**B**) Body weight (g), mean ± SEM. (**C**) H&E staining, Masson staining, and immunohistochemical staining of TK1. (**D**) Hydroxyproline content was quantified in lung tissues (*n* = 4), with *P < 0.05, **P < 0.01, and ***P < 0.001 indicating significance. (**E**-**F**) Lactate concentrations were measured in the alveolar lavage fluid and lung tissue of mice in the indicated groups (*n* = 4), with **P < 0.01 and ***P < 0.001. (**G**) Expression levels of Fibronectin, Collagen I, α-SMA, TK1, and PFKFB3 were assessed by western blot analysis. GAPDH served as a loading control. The experiments were repeated three times with consistent results. (**H**-**I**) Glucose consumption and lactate levels were measured in primary fibroblast cultures treated with Vitamin D or 3PO (*n* = 4), with *P < 0.05 and **P < 0.01. For **B**, a 2-way repeated measures ANOVA was used. For **D**, **E**, **F**, **H**, and **I**, one-way ANOVA with the Bonferroni multiple comparisons test was applied. Data are expressed as mean ± SD. Source data are provided in the Source data file.

To further explore the roles of Vitamin D and 3PO in pulmonary fibrosis, a bleomycin-induced murine model was employed. Mice were given a single intratracheal dose of bleomycin, followed by treatments with Vitamin D (10 µg/kg/3d), 3PO (20 mg/kg/3d), or their combination over a period of 21 days (Fig. 7A). Vitamin D and 3PO therapies resulted in a reduction of body weight loss compared to the bleomycin-exposed group (Fig. 7B). Histopathological evaluation using H&E and Masson’s trichrome staining revealed that bleomycin significantly worsened alveolar structural destruction and induced notable collagen deposition. Conversely, administration of Vitamin D or 3PO markedly alleviated these pathological alterations, with combined therapy demonstrating the most substantial improvement (Fig. 7C and S8A). Immunohistochemical analyses confirmed that both Vitamin D and 3PO significantly decreased TK1 expression in pulmonary fibrosis (Fig. 7C). Consistent with histological observations, hydroxyproline content was markedly reduced following treatment compared to the bleomycin group (Fig. 7D). Additionally, interventions with Vitamin D or 3PO suppressed lactate concentrations in BALF and lung tissues (Fig. 7E, F). Western blot analyses revealed that all treatments considerably downregulated fibrotic markers, including Fibronectin, Collagen I, and α-SMA, as well as glycolytic regulators TK1 and PFKFB3 (Fig. 7G and S8B). Furthermore, glucose uptake and lactate production in primary fibroblasts were significantly increased after bleomycin administration; however, treatments with Vitamin D, 3PO, or their combination effectively mitigated this metabolic imbalance (Fig. 7H, I). Moreover, analyses of inflammatory cytokines in BALF and lung tissues indicated that bleomycin induced substantial increases in IL-1β, IL-6, and TNF-α levels, which were attenuated by all treatments (Fig. S8C-G). Additionally, qRT-PCR analysis demonstrated a prominent elevation of MMP2 and MMP8, alongside a decrease in MMP7 levels, in the bleomycin-treated group relative to controls, paralleling observations in silica-induced pulmonary fibrosis (Fig. S8H-J). Both Vitamin D and 3PO treatments successfully normalized the expression of matrix metalloproteinases. These findings suggest that treatment with Vitamin D and 3PO mitigates bleomycin-induced pulmonary fibrosis.

## Discussion

Pulmonary fibrosis occurs in a variety of clinical settings and significantly contributes to morbidity and mortality [27]. However, current therapeutic interventions demonstrate limited effectiveness in improving patient outcomes. The proliferation and activation of ECM-producing myofibroblasts are crucial to the progression of pulmonary fibrosis, though the underlying mechanisms are not yet fully understood. Consequently, identifying targets that drive the abnormal proliferation and differentiation of myofibroblasts, or that enhance profibrotic gene expression, could be highly beneficial for the development of therapies aimed at treating pulmonary fibrosis. This study employed the MRC-5 human fetal lung fibroblast cell line as an in vitro model for pulmonary fibrosis, primarily due to the limited availability of human lung tissue specimens. MRC-5 cells exhibit key profibrotic phenotypes, including cellular proliferation, differentiation, and ECM synthesis. To enhance the translational significance of the findings, primary fibroblasts isolated from murine sources were also incorporated to simulate in vivo conditions. Additionally, we established a cellular model of fibroblast activation induced by TGF-β1, along with murine pulmonary fibrosis models induced by silica or bleomycin, to investigate the fundamental molecular mechanisms underlying pulmonary fibrosis. While this comprehensive approach strengthens our conclusions, future research utilizing primary human lung fibroblasts will be imperative to fully assess translational potential.

Aberrant cellular metabolism has been shown to participate in many pathologic processes. Several studies have demonstrated that glycolytic reprogramming is critical to lung myofibroblast differentiation by providing energy, promoting cell proliferation, and increasing extracellular matrix synthesis [28, 29]. Moreover, the metabolite lactic acid has also been observed as an important mediator of myofibroblast differentiation via a pH-dependent activation of TGF-β1 [30]. Our data similarly indicate that TGF-β1, which is known to activate fibroblasts, also enhances glycolysis. Notably, we observed an increase in the protein levels of Fibronectin, Collagen I, and α-SMA in fibroblasts treated with TGF-β1, accompanied by elevated lactate production, glucose uptake, ATP levels, and ECAR. In contrast, the glycolysis inhibitor 2-DG effectively inhibited the increase in glycolysis and fibrosis marker levels triggered by TGF-β1. Moreover, lactate production was also elevated in the lungs following silica or bleomycin treatment. These findings offer a new theoretical basis for targeting fibroblast glycolysis in the treatment of pulmonary fibrosis.

Previous studies have demonstrated that specific glycolytic inhibitors can inhibit fibroblast activation, thereby reducing fibrosis. Specifically, 3PO (an inhibitor of PFKFB3) effectively attenuates lung fibroblast differentiation into myofibroblasts, thereby reducing fibrotic progression [31]. Administering dichloroacetate (DCA) or shikonin could reduce renal fibrosis in a murine model of unilateral ureteral obstruction (UUO) by targeting glycolytic enzymes [32]. Similarly, aerobic glycolysis inhibitors shikonin and 2-deoxyglucose attenuate UUO-induced mouse renal fibrosis and TGF-β1-stimulated myofibroblast activation [33]. In summary, although glycolysis inhibitors have demonstrated potential antifibrotic effects in cellular and animal studies, many of these inhibitors also show significant cytotoxicity, leading to concerns about their safety in humans. Exploring milder alternatives could be a practical approach to address this issue.

Vitamin D is recognized as a biologically active substance that has various effects beyond just regulating calcium and phosphorus levels in the body. Several epidemiological studies indicate that the concentration of Vitamin D in the serum may be linked to the severity of fibrotic diseases, including pulmonary fibrosis [34–36]. An experimental study has demonstrated that Vitamin D3 may serve as a potential antifibrotic agent in chronic kidney disease through activating the Vitamin D receptor and inhibiting the TGF-β1/Smad3 signaling pathway [37]. Another study indicated that Vitamin D exerts a regulatory effect by downregulating the expression of fibrogenic mediators and ECM through the intervention of the local pulmonary renin-angiotensin system (RAS) [38]. Furthermore, Vitamin D can mitigate renal interstitial fibrosis through a multifaceted approach, including inhibiting the RAAS, reducing inflammation, and suppressing the epithelial-to-mesenchymal transition [36]. However, the relationship between fibroblast metabolic reprogramming and Vitamin D remains largely unexplored. In our study, we found that Vitamin D significantly inhibited the expression of profibrotic markers and the proliferation of lung fibroblasts stimulated by TGF-β1. Interestingly, markers associated with glycolysis—such as lactate production, glucose uptake, ATP levels, and ECAR—were also down-regulated following Vitamin D treatment in both MRC-5 cells and primary mouse pulmonary fibroblasts. This suggests that Vitamin D may play a role in regulating fibroblast activation through its effects on glycolysis.

The Vitamin D receptor (VDR), a ligand-activated transcription factor, serves as the primary mediator of the genomic actions of Vitamin D. Increasing evidence suggests a canonical role for VDR signaling in metabolic reprogramming, including the regulation of glycolytic pathways, as well as in suppressing pro-fibrotic fibroblast activation. Studies across various fibroblast populations have demonstrated that genetic or pharmacological activation of VDR inhibits proliferation and extracellular matrix production, while deficiency in VDR exacerbates these processes [39, 40]. Additionally, VDR ablation has been shown to enhance insulin signaling and amplify glycolysis and lipogenesis, underscoring its extensive influence on cellular metabolism [41, 42]. Our previous investigations align with this framework, confirming that the Vitamin D/VDR axis attenuates fibroblast activation and glycolysis through the suppression of the STAT3/HK2 signaling pathway [43]. Nonetheless, despite the acknowledged role of VDR in regulating fibroblast phenotype and metabolism, the complete spectrum of its downstream targets remains inadequately elucidated.

Next, we explored the potential targets of Vitamin D and confirmed TK1 as a functional downstream effector of Vitamin D via RNA-seq analysis and experimental studies. TK1 exhibited elevated expression in fibroblasts following TGF-β1 treatment, evident at both the mRNA and protein levels. Additionally, a negative correlation was observed between Vitamin D levels and TK1 expression. The knockdown of VDR significantly elevated the levels of both mRNA and protein expression of TK1, suggesting that Vitamin D potentially influences TK1 expression via the regulation of VDR. Furthermore, the knockdown of TK1 effectively negated the increase in glycolysis and fibrosis marker levels induced by TGF-β1, while the overexpression of TK1 restored this increase. TK1 is an enzyme in the DNA salvage pathway that is involved in cellular proliferation through the recycling of thymidine nucleotides [44]. As a member of the thymidine kinase enzyme family, TK1 has been consistently demonstrated in recent research to be markedly upregulated across multiple cancer phenotypes [45, 46]. Overexpression of TK1 has a profound aggravating effect on the progression of hepatocellular carcinoma, acting through both enzyme-dependent and -independent mechanisms [47]. Interestingly, recent evidence suggests that stabilized TK1 can promote glycolysis independently of its enzymatic activity, providing novel insights into TK1’s non-enzymatic roles in cancer metabolism [48]. While TK1 upregulation is a hallmark of cancer progression, its role in fibrosis has been underexplored. Here, we confirmed that TK1 enhances fibroblast activation and glycolysis by regulating the well-known glycolysis trigger, PFKFB3. The protein expression of PFKFB3 was elevated in fibroblasts after TGF-β1 treatment or TK1 overexpression. On the contrary, the expression of PFKFB3 was down-regulated by small interfering RNA (siRNA)-mediated TK1 knockdown. Besides, TK1 overexpression induced fibroblast activation and glycolysis, whereas 3PO, a well-established inhibitor of PFKFB3, reversed its effects.

Additionally, we administered Vitamin D and the compound 3PO, either separately or in combination, to the experimental mice. Both Vitamin D and 3PO exhibited notable antifibrotic properties independently; however, their synergistic interaction produced a markedly enhanced antifibrotic effect, resulting in a greater reduction in the severity of fibrosis compared to the effects of either agent alone. Importantly, the present investigation demonstrates that Vitamin D inhibits bleomycin-induced pulmonary fibrosis by downregulating the TK1/PFKFB3 pathway, in alignment with findings in a silicosis model. Furthermore, Vitamin D alleviates inflammation and restores MMP balance, thereby supporting tissue remodeling. Considering that the bleomycin model replicates essential features of human idiopathic pulmonary fibrosis (IPF), these findings highlight the potential of Vitamin D as a therapeutic agent for fibrotic lung diseases. Recent studies indicate that TK1 promotes tumor growth beyond its role in dTMP synthesis, including enzyme-independent mechanisms such as stabilizing PRMT1 to enhance glycolysis and proliferation in hepatocellular carcinoma [47]. Upstream growth factors like EGF and IGF-1 augment TK1 stability via ERK phosphorylation and deubiquitination, thereby amplifying DNA synthesis and glycolysis in cancer cells [48]. This implies a broader functional role for TK1 in cellular metabolism. In conclusion, our study is the first to demonstrate that Vitamin D can exert potent antifibrotic effects by modulating the TK1/PFKFB3 signaling axis.

Although the TK1/PFKFB3 signaling pathway was identified as a mediator of Vitamin D’s effects on fibroblast activation and glycolysis in the present study, we cannot exclude the possibility that Vitamin D alleviates fibrogenesis via other molecular mechanisms. Moreover, TGF-β1 was observed to enhance TK1 expression; however, few reports exist on the underlying molecular mechanism between TGF-β1 and TK1. Remarkably, our results showed that PFKFB3 was negatively correlated with the expression of TK1 at the protein level but not at the mRNA level. However, the precise molecular mechanisms underlying this post-transcriptional regulation remain unclear. Furthermore, our results indicate that the combined administration of Vitamin D and 3PO results in a reduction of various inflammatory mediators, suggesting that these agents may attenuate pulmonary fibrosis through multiple mechanistic pathways. Nonetheless, the precise molecular mechanisms underlying these effects warrant further investigation. Consequently, additional studies are needed to elucidate additional targets of Vitamin D and the interactions between TK1 and PFKFB3.

## Conclusions

**Fig. 8:**
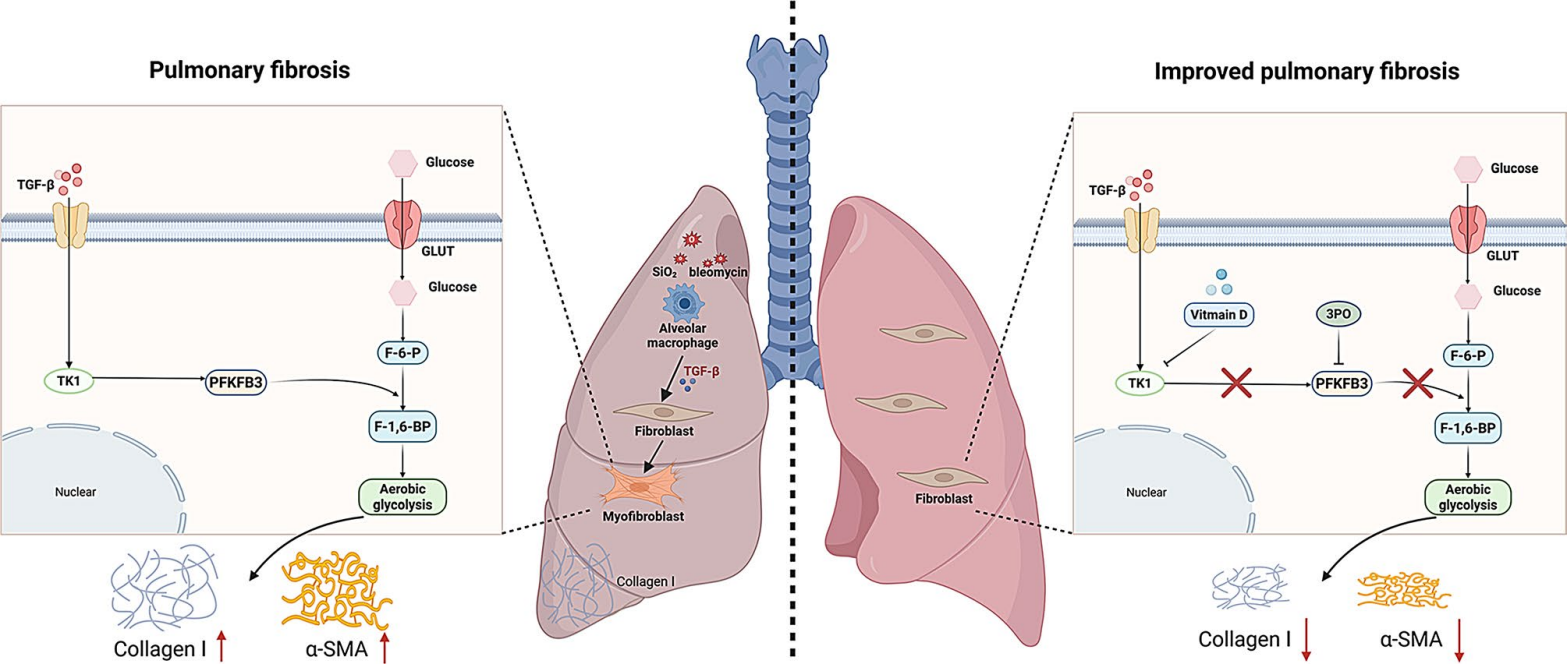
Schematic illustration represents that Vitamin D regulates fibroblast glycolysis and activation by inhibiting TK1/PFKFB3 signaling pathway during pulmonary fibrogenesis

In conclusion, our study revealed a novel Vitamin D-mediated mechanism that underlies the pathology of pulmonary fibrosis. Specifically, Vitamin D exerted antifibrotic effects by disrupting the interaction between the TK1 and PFKFB3-driven glycolysis, subsequently inhibiting fibroblast activation in pulmonary fibrosis (Fig. 8). These findings not only provide valuable insights into the molecular mechanisms underlying fibroblast activation and fibrosis but also suggest that the TK1/PFKFB3 signaling pathway may serve as a promising therapeutic target for the management of fibrotic diseases.

## Supplementary Information

Below is the link to the electronic supplementary material.

Supplementary Material 1

Supplementary Material 2

## Data Availability

All datasets used and/or analyzed supporting the conclusions are available from the corresponding author upon reasonable request.

## Abbreviations

α-SMA: alpha-smooth muscle actin
BALF: bronchoalveolar lavage fluid
DMSO: dimethyl sulfoxide
ECAR: extracellular acidification rate
ECM: excessive extracellular matrix
ELISA: enzyme-linked immunosorbent assay
EMT: epithelial-mesenchymal transition
FMT: fibroblast-myofibroblast transition
H&E: hematoxylin and eosin staining
IL-1β: interleukin-1β
IL-6: interleukin-6
PBS: phosphate-buffered saline
PFKFB3: 6-phosphofructo-2-kinase/fructose-2,6-bisphosphatase 3
siRNA: small interfering RNA
TGF-β: transforming growth factor-β
TK1: Thymidine Kinase 1
TNF-α: tumor necrosis factor-alpha
VDR: vitamin D receptor
2-DG: 2-Deoxy-d-glucose
3PO: 3-[3-pyridinyl]-1-[4-pyridinyl]-2-propen-1-one
