# Supplementary material for: Vitamin D attenuates the progression of pulmonary fibrosis via inhibiting thymidine kinase 1/PFKFB3-driven glycolysis
Source: J Transl Med. 2026 Apr 22;24:571. doi: 10.1186/s12967-025-07298-1 (PMC13104274; doi:10.1186/s12967-025-07298-1)
Supplement: Supplementary file 1 — Supplementary Material 1 [file 12967_2025_7298_MOESM1_ESM.pdf]

Vitamin D attenuates the progression of pulmonary fibrosis via inhibiting Thymidine Kinase 1/PFKFB3-driven glycolysis

Huanyu Yang[1†], Li Zhang[2†], Mengjia Han[1], Keye Zhu[3], Xianghan Guo[3], Wenkang Yang[1], Qi Xu[1*]

[1] School of Public Health, Binzhou Medical University, Yantai 264003, China

[2] School of Health Management, Binzhou Medical University, Yantai 264003, China

[3] Second School of Clinical Medicine, Binzhou Medical University, Yantai 264003, China

*Correspondence to: School of Public Health, Binzhou Medical University, NO 346, Guanhai Road, Yantai, Shandong Province 264003, China

E-mail address: xuqi@bzmc.edu.cn; xuqi9876@126.com

†Huanyu Yang and Li Zhang contributed equally to this work.

Figure S1

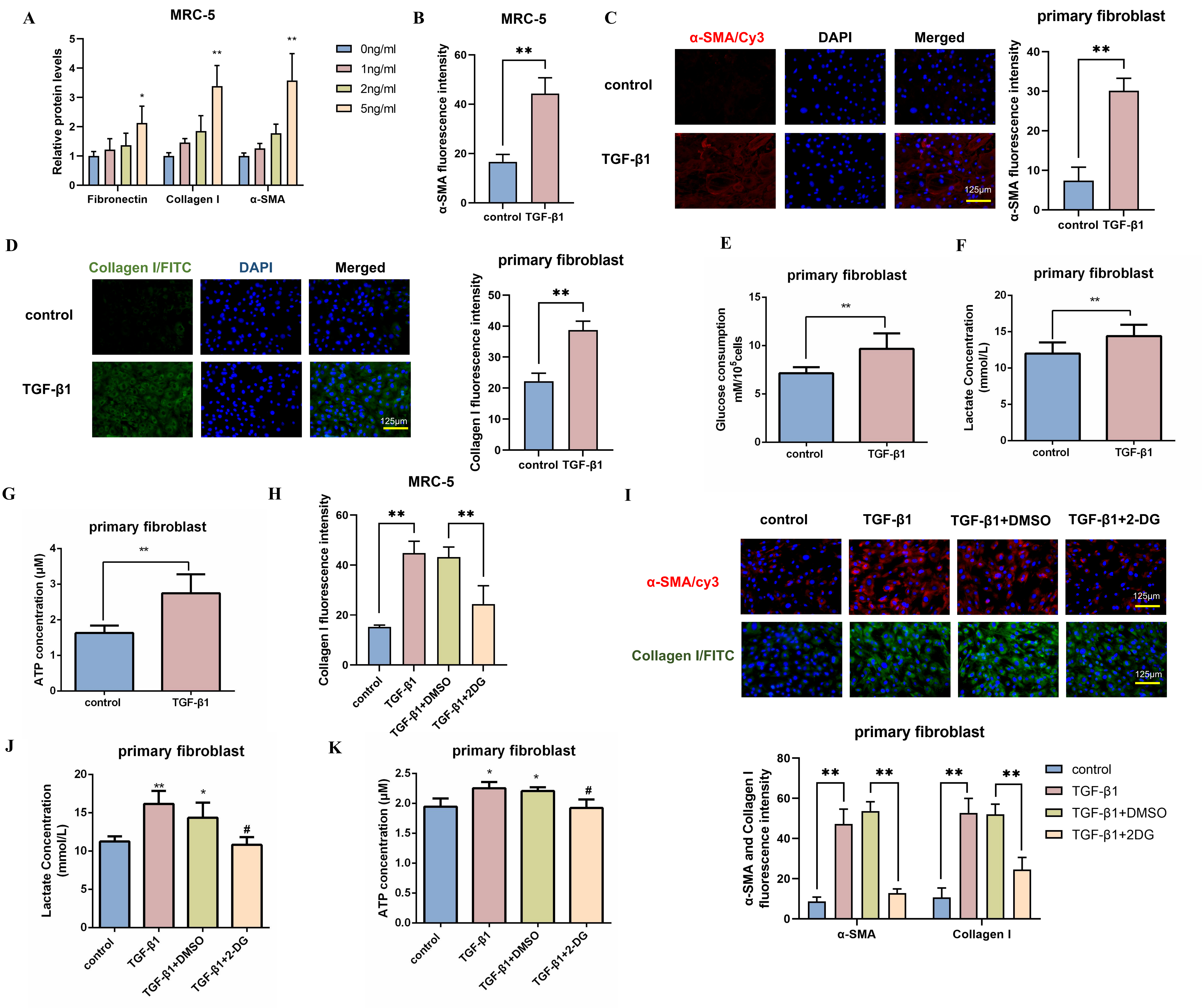

Figure S1. Enhanced glycolysis contributes to pulmonary fibroblast activation. (A) Densitometric analysis of Fibronectin, Collagen I, and α-SMA in MRC-5 cells (n=3), with * P<0.05, ** P<0.01 vs. control group. (B) Fluorescence intensity of α-SMA in MRC-5 cells treated with TGF-β1 for 48 h (n=3), with and ** P<0.01 vs. control group. (C-D) Mouse primary lung fibroblasts were subjected to staining utilizing a primary antibody specific to the myofibroblast marker α-SMA (red) and Collagen I (green). Bar chart showing the mean fluorescence intensity of α-SMA and Collagen in mouse primary lung fibroblasts (n=3), with ** P<0.01 vs. control group. The nuclei were stained with the blue fluorescent DAPI. Scale bar = 125 μm. (E-G) Glucose consumption, lactate concentration, and ATP concentration were detected in primary fibroblasts treated with TGF-β1 for 48 h (n = 3), **P < 0.01. (H) The mean fluorescence intensity of Collagen I in MRC-5 cells treated with 2-DG at 10 mM for 1 hour, followed by exposure to TGF-β1 for 48 hours (n=3), with ** P<0.01. (I) Immunofluorescence staining images and mean fluorescence intensity for α-SMA and Collagen I in primary fibroblasts pretreated with 2-DG at 10 mM for 1 h, then exposed to TGF-β1 for 48 h, were performed to evaluate cell proliferative ability (n=3), with ** P<0.01. Red represents α-SMA staining; green represents Collagen I staining; blue represents nuclear DNA staining by DAPI. Scale bar = 125μm. (J-K) Lactate concentration and ATP concentration were detected in primary fibroblasts for the indicated groups (n = 3), *P < 0.05, **P < 0.01 vs. the control group, and #P < 0.05 vs. TGF-β1+DMSO group. For A and I, 2-way ANOVA was used. For B, C, D, E, F, and G, a two-tailed t-test was used. For H, J, and K, one-way ANOVA was used with the Bonferroni multiple comparisons test. Data are presented as mean ± SD. Source data are provided as a Source data file.

Figure S2

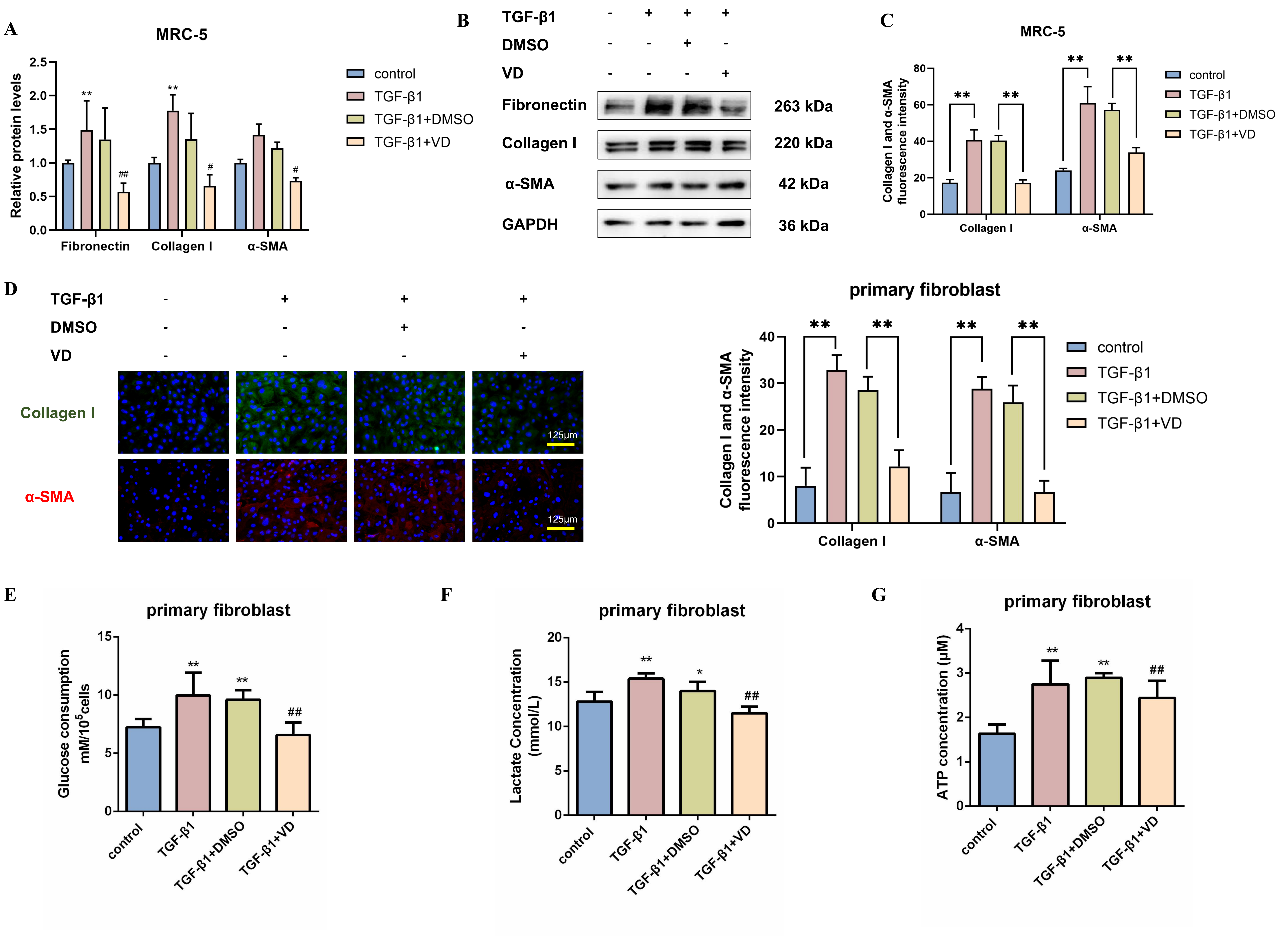

Figure S2. Vitamin D exerts anti-fibrotic effects by reducing glycolysis. (A) Densitometric analysis of Fibronectin, Collagen I, and α-SMA in MRC-5 cells treated with Vitamin D at 100 nM for 24 hours and then exposed to TGF-β1 5ng/ml for 48 hours (n=3), with ** P<0.01 vs. control group, and #P < 0.05, ##P < 0.01 vs. TGF-β1+DMSO group. (B) Western blot analysis of Fibronectin, Collagen I, and α-SMA in primary fibroblasts was treated with vitamin D (100 mM). (C) Mean fluorescence intensity of Collagen I and α-SMA in MRC-5 cells (n=3), with ** P<0.01. (D) Immunofluorescence staining analysis and mean fluorescence intensity of Collagen I and α-SMA in primary fibroblasts (n=3), with ** P<0.01. Scale bar = 125 μm. (E-G) Glucose consumption, lactate concentration, and ATP concentration were detected in primary fibroblasts for the indicated groups (n = 3), *P < 0.05, **P < 0.01 vs. the control group, and ##P < 0.01 vs. TGF-β1+DMSO group. For A, C, and D, 2-way ANOVA was used. For E, F, and G, one-way ANOVA was used. Data are presented as mean ± SD. Source data are provided as a Source data file.

Figure S3

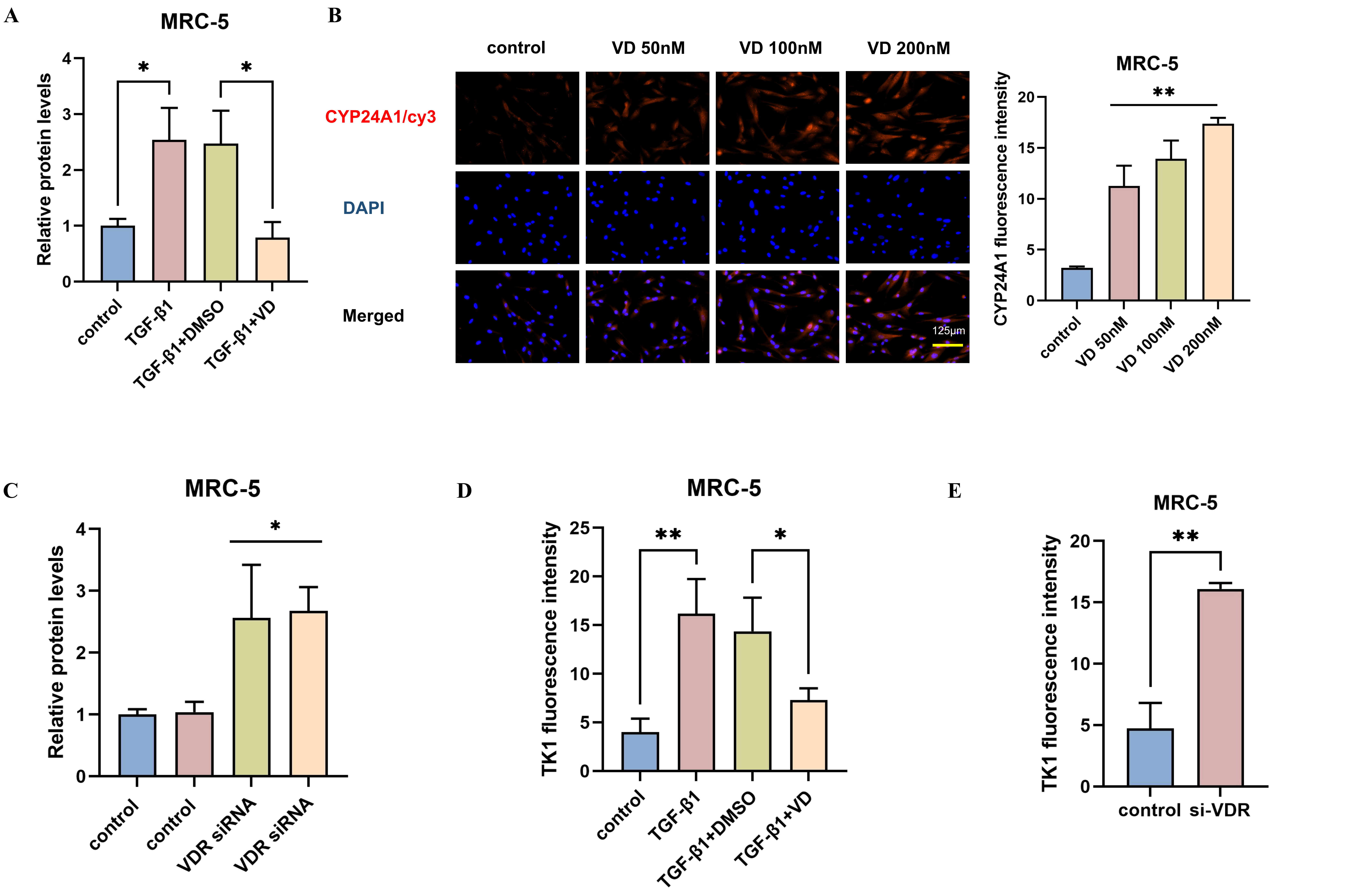

Figure S3. TK1 is a downstream target of Vitamin D in lung fibroblasts. (A) Densitometric analysis of TK1 in MRC-5 cells treated with Vitamin D at 100 nM for 24 hours, followed by exposure to TGF-β1 at 5 ng/ml for 48 hours (n=3), with * P<0.05. (B) Immunofluorescence staining analysis and mean fluorescence intensity of CYP24A1 in MRC-5 cells treated with Vitamin D at 50, 100, 200 nM for 24 hours (n=3), with** P<0.01. Scale bar = 125 μm. (C) Densitometric analysis of TK1 in MRC-5 cells treated with VDR siRNA for 96 hours (n=3), with *P<0.05. (D-E) Mean fluorescence intensity of TK1 in MRC-5 cells (n=3), with * P<0.05, ** P<0.01. For A, B, C, and D, one-way ANOVA was used. For E, a two-tailed t-test was used. Data are presented as mean ± SD. Source data are provided as a Source data file.

Figure S4

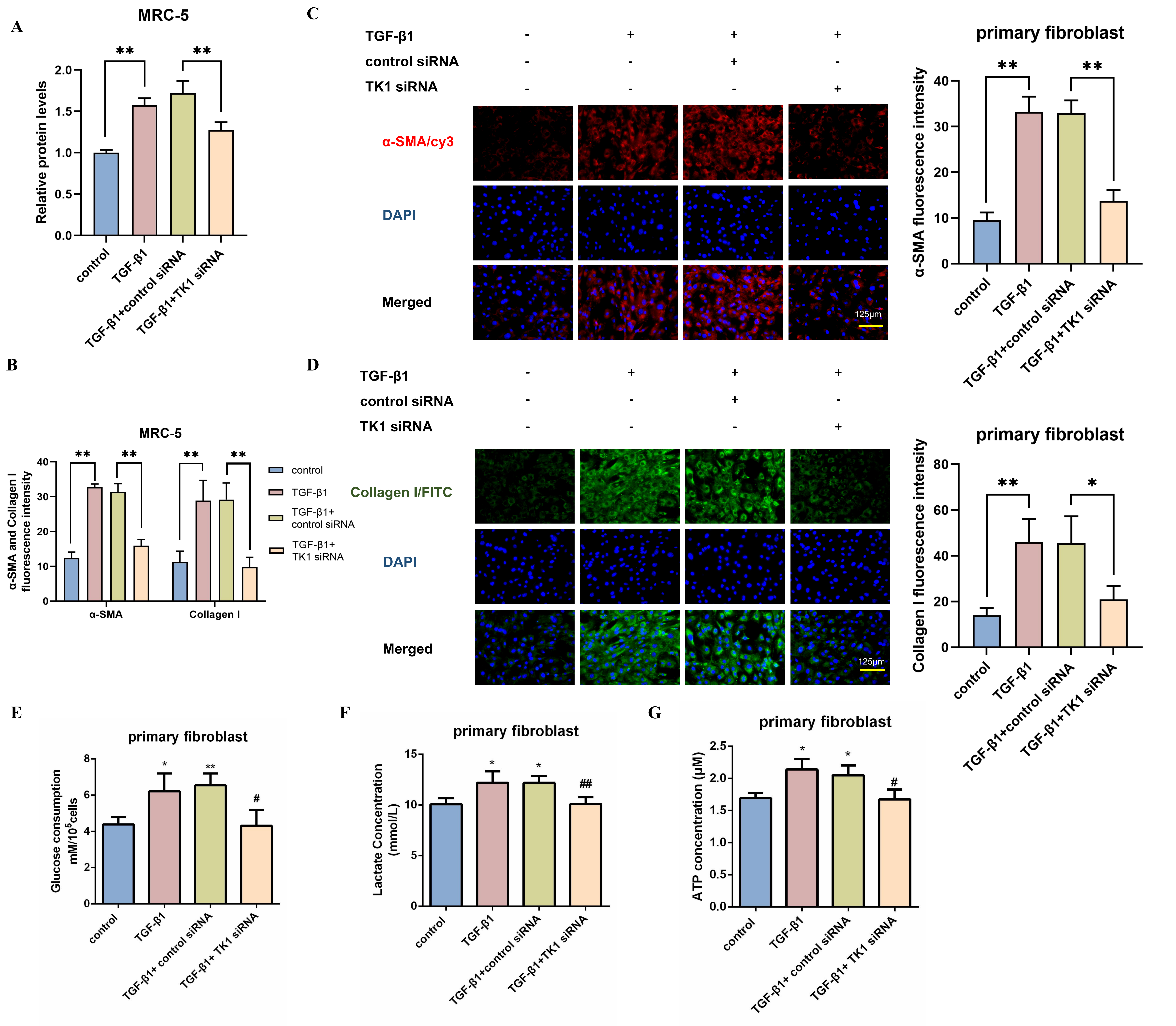

Figure S4. TK1 is involved in fibroblast activation and glycolysis. (A) Densitometric analysis of TK1 in MRC-5 cells treated with TK1 or control siRNA for 24 hours, followed by exposure to TGF-β1 at 5 ng/ml for 48 hours (n=3), with ** P<0.01. (B) Mean fluorescence intensity of α-SMA and Collagen I in MRC-5 cells (n=3), with ** P<0.01. (C-D) The expression of α-SMA (red) and Collagen I (green) was assessed via immunofluorescence staining to show fibroblast activation. Bar chart showing the mean fluorescence intensity of α-SMA and Collagen in mouse primary lung fibroblasts (n=3), with * P<0.05, ** P<0.01. Scale bar = 125μm. (E-G) Glucose consumption, lactate concentration, and ATP concentration were detected in primary fibroblasts for the indicated groups (n = 3), with *P < 0.05, **P < 0.01 vs. the control group, #P < 0.05, and ##P < 0.01 vs. TGF-β1+control siRNA group. For A, C, D, E, F, and G, one-way ANOVA was used. For B, 2-way ANOVA was used. Data are presented as mean ± SD. Source data are provided as a Source data file.

Figure S5

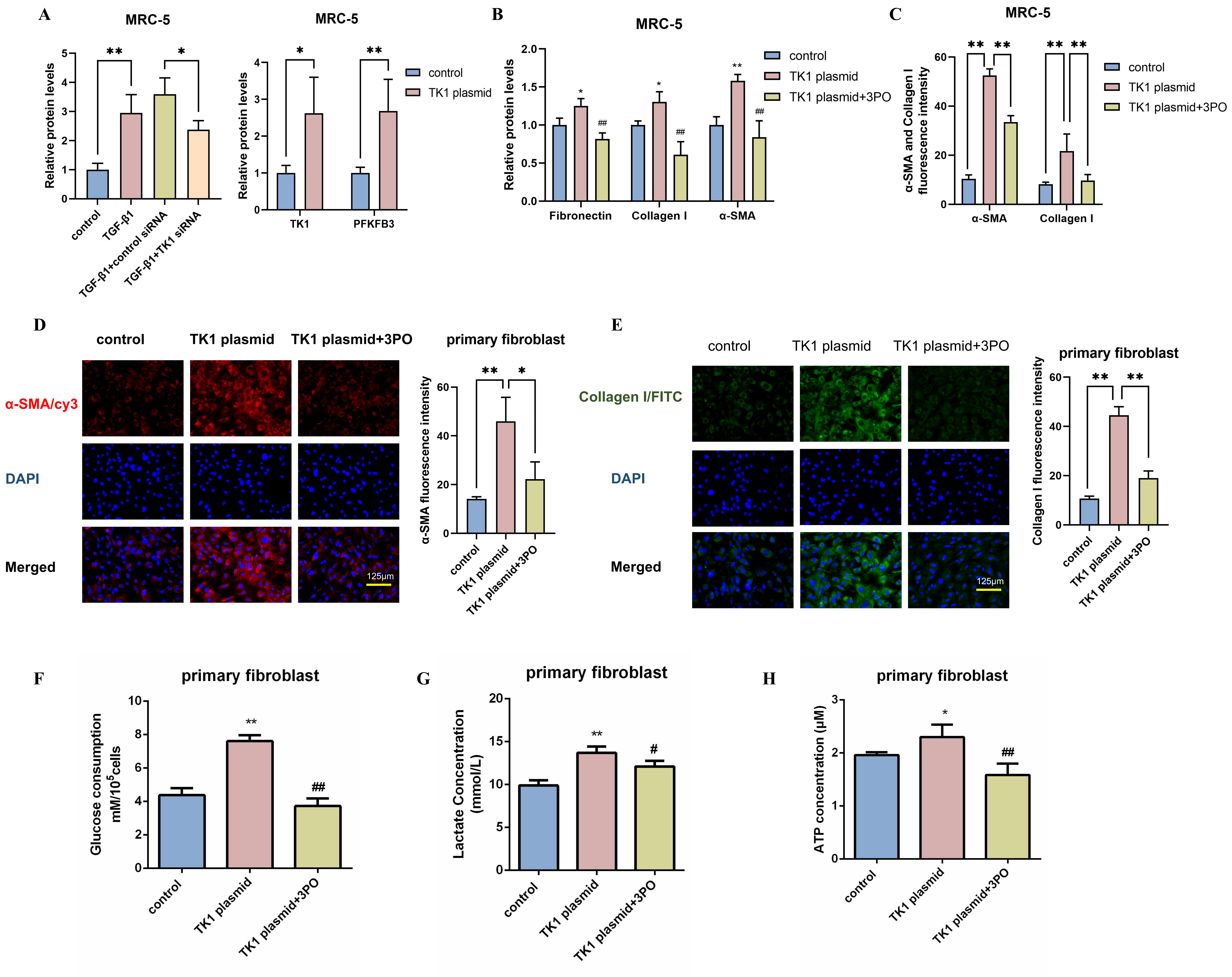

Figure S5. PFKFB3 mediates the function of TK1 to regulate fibroblast activation and glycolytic reprogramming. (A) Left: Densitometric analysis of PFKFB3 in MRC-5 cells treated with TK1 or control siRNA for 24 hours, followed by exposure to TGF-β1 at 5 ng/ml for 48 hours (n=3), with * P<0.05, ** P<0.01. Right: Densitometric analysis of TK1 and PFKFB3 in MRC-5 cells treated with TK1 plasmid for 96 hours (n=3), with * P<0.05, ** P<0.01. (B) Densitometric analysis of Fibronectin, Collagen I, and α-SMA in MRC-5 cells treated with 3PO for 1 hour, then exposed to TK1 plasmid for 96 hours (n=3), with * P<0.05, ** P<0.01 vs. the control group, and ##P < 0.01 vs. TK1 plasmid group. (C) Mean fluorescence intensity of α-SMA and Collagen I in MRC-5 cells (n=3), with ** P<0.01. (D-E) Immunofluorescence staining and mean fluorescence intensity of α-SMA (red) and Collagen I (green) in mouse primary lung fibroblasts treated with TK1 plasmid or 3PO (n=3), with * P<0.05, ** P<0.01. Scale bar = 125μm. (F-H) Glucose consumption, lactate concentration, and ATP concentration were detected in primary fibroblasts for the indicated groups (n = 3), with *P < 0.05, **P < 0.01 vs. the control group, #P < 0.05, and ##P < 0.01 vs. TK1 plasmid group. For A (left), D, E, F, G, and H, one-way ANOVA was used. For A (right), B, and C, 2-way ANOVA was used. Data are presented as mean ± SD. Source data are provided as a Source data file.

Figure S6

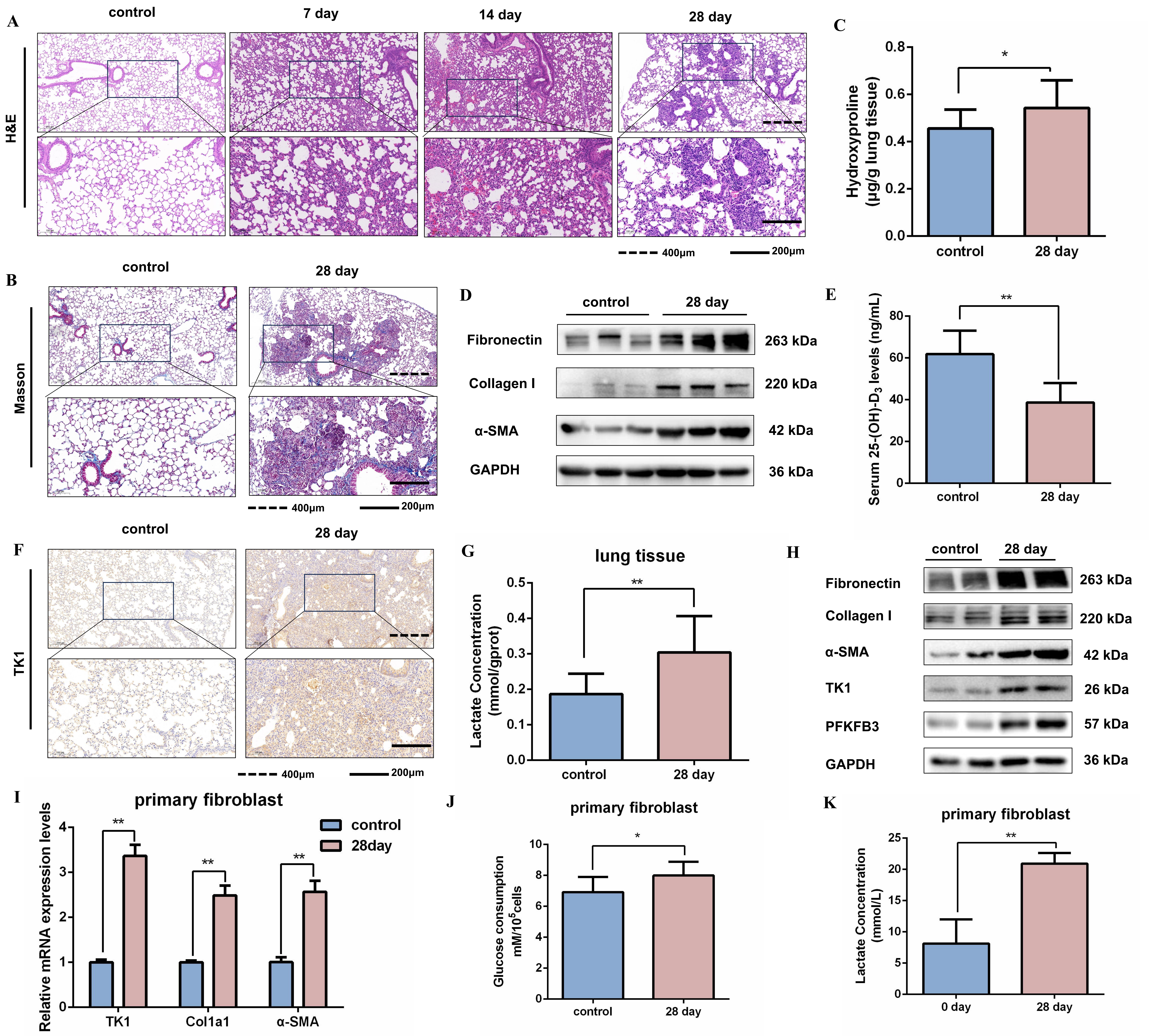

Figure S6. Silica-induced pulmonary fibrosis is linked to vitamin D and glycolytic dysregulation. (A-B) The C57BL/6 mice were sacrificed on days 7, 14, and 28 after intratracheal instillation of silica suspended in saline solution. Representative images of H&E staining for histology (A) and Masson's trichrome staining for collagen deposition (B) in mouse lung tissues (n = 4 mice per group). (C) Hydroxyproline content of the lung tissues was used to assess the degree of collagen deposition (n = 4 mice per group), with $*P < 0.05$. (D) Western blot analysis evaluated the protein expression of Fibronectin, Collagen I, and α-SMA in murine lung tissues. GAPDH served as the internal loading control for the assays. The experiments were repeated three times and the results were similar. (E) The quantification of serum 25-(OH)-D3 was assessed in lung tissues subjected to silica exposure over 28 days (n = 4), $**P < 0.01$. (F) Representative images of immunohistochemical staining of TK1 in lung sections. (G) Lactate concentration was detected in lung tissues exposed to silica for 28 days (n = 4), with $**P < 0.01$. (H) Fibronectin, Collagen I, α-SMA, TK1, and PFKFB3 protein levels in primary lung fibroblasts. GAPDH was used as a loading control. The experiments were repeated three times and the results were similar. (I) TK1, Collagen I, α-SMA mRNA expression in primary lung fibroblasts (n = 4), with $**P < 0.01$. (J-K) Glucose consumption and lactate levels were determined (n = 4), with $*P < 0.05$, $**P < 0.01$ vs. the control group. For C, E, G, J, and K, a two-tailed t-test was used. For I, 2-way ANOVA was used. Data are presented as mean ± SD. Source data are provided as a Source data file.

Figure S6

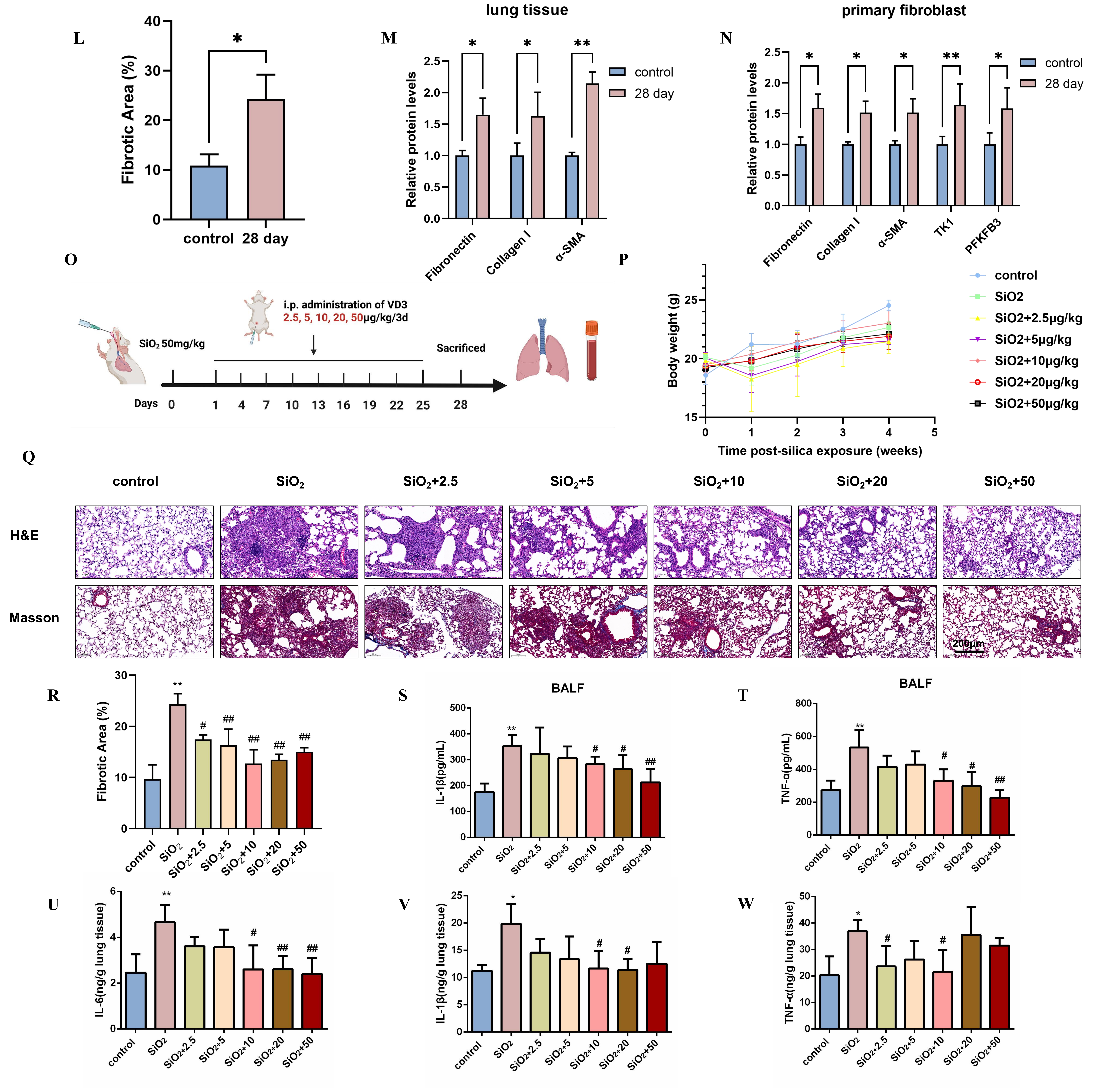

Figure S6. Silica-induced pulmonary fibrosis is linked to vitamin D and glycolytic dysregulation. (L)The severity of fibrosis was evaluated by the Masson's trichrome staining (n=4), with * P<0.05. (M) Densitometric analysis of Fibronectin, Collagen I, and α-SMA in mouse lung tissues treated with silica for 28 days (n=3), with * P<0.05, ** P<0.01. (N) Densitometric analysis of Fibronectin, Collagen I, α-SMA, TK1, and PFKFB3 in mouse primary lung fibroblasts treated with silica for 28 days (n=3), with * P<0.05, ** P<0.01. (O) Strategy for administering vitamin D in a silica-induced pulmonary fibrosis murine model. (P) Body weight (g), mean ± SEM. (Q-R) H&E staining, Masson's trichrome staining, and the calculated fibrotic area (n=4), with ** P<0.01 vs. the control group, and #P < 0.05, ##P < 0.01 vs. SiO_2 group. (S-W) Concentration of IL-6, IL-1β, and TNF-α in BALF and lung tissues by ELISA (n = 4), with *P < 0.05, **P < 0.01 vs. the control group, and #P < 0.05, ##P < 0.01 vs. SiO_2 group. For L, a two-tailed t-test was used. For M and N, 2-way ANOVA was used. For R, S, T, U, V, and W, one-way ANOVA was used. Data are presented as mean ± SD. Source data are provided as a Source data file.

Figure S7

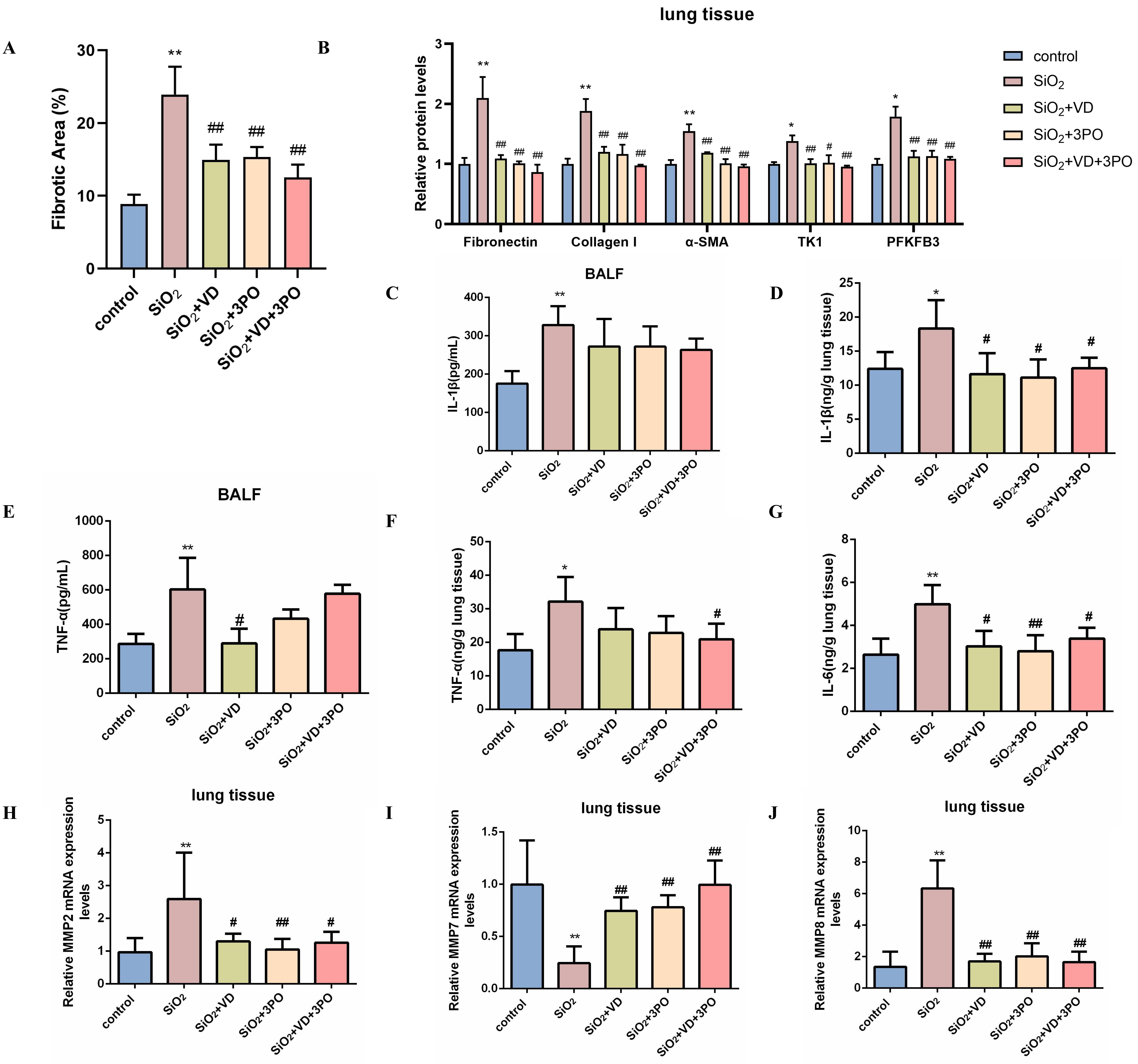

Figure S7. Combined Vitamin D and 3PO alleviates silica-induced pulmonary fibrosis *in vivo*. (A)The severity of fibrosis was evaluated by the Masson's trichrome staining (n=4), with ** P<0.01 vs. the control group, and ##P < 0.01 vs. SiO_2 group. (B) Densitometric analysis of Fibronectin, Collagen I, α-SMA, TK1, and PFKFB3 in mouse lung tissues (n=3), with * P<0.05, ** P<0.01 vs. the control group, and #P < 0.05, ##P < 0.01 vs. SiO_2 group. (C-G) Concentration of IL-6, IL-1β, and TNF-α in BALF and lung tissues by ELISA (n = 4), with *P < 0.05, **P < 0.01 vs. the control group, and #P < 0.05, ##P < 0.01 vs. SiO_2 group. (H-J) Relative mRNA expression of MMP2, MMP7, and MMP8 in lung tissues (n = 4), with **P < 0.01 vs. the control group, and #P < 0.05, ##P < 0.01 vs. SiO_2 group. For A, C, D, E, F, G, H, I, and J, one-way ANOVA was used. For B, 2-way ANOVA was used. Data are presented as mean ± SD. Source data are provided as a Source data file.

Figure S8

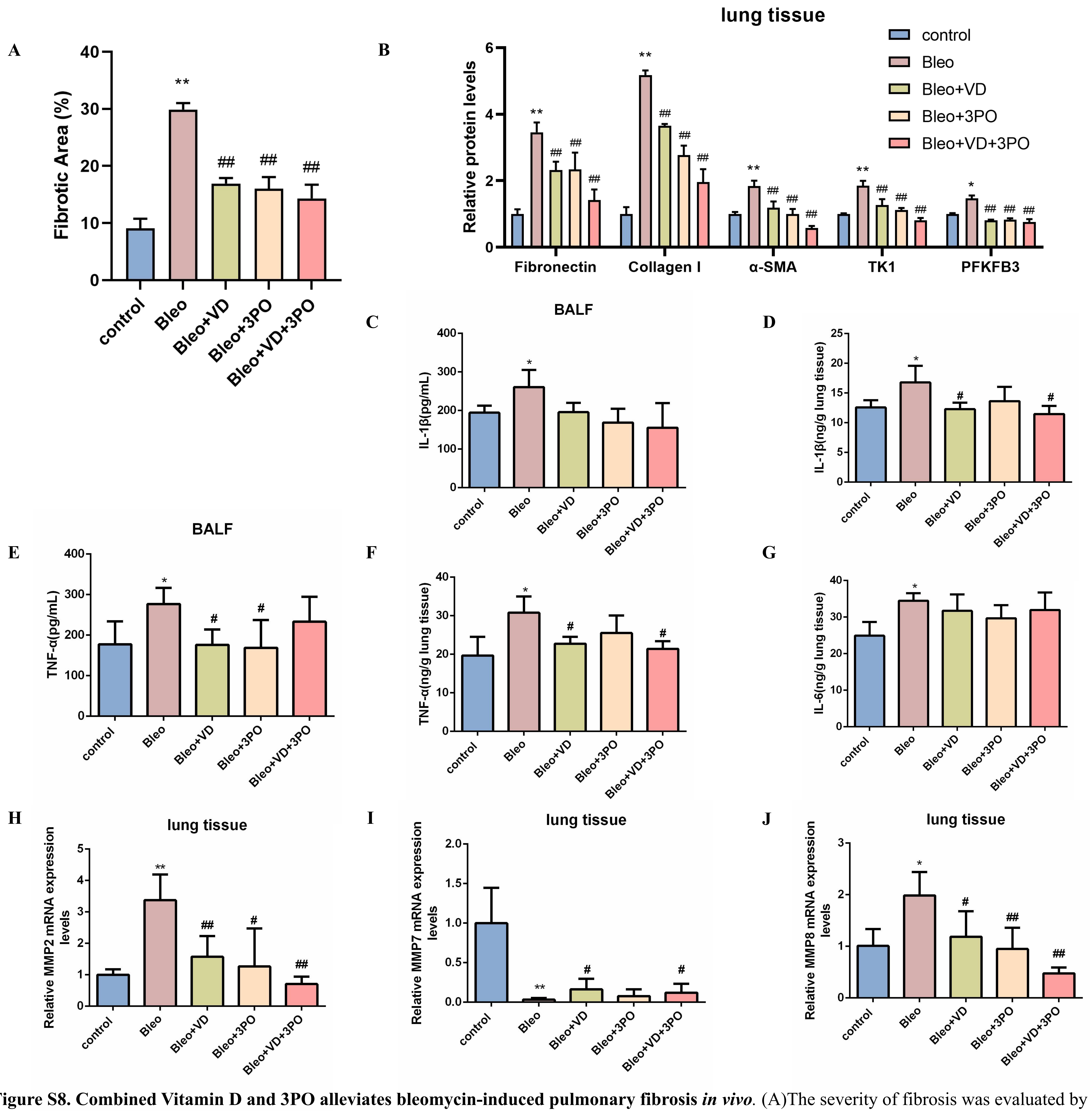

Figure S8. Combined Vitamin D and 3PO alleviates bleomycin-induced pulmonary fibrosis *in vivo*. (A)The severity of fibrosis was evaluated by the Masson's trichrome staining (n=4), with ** $P<0.01$ vs. the control group, and ##$P < 0.01$ vs. the bleomycin group. (B) Densitometric analysis of Fibronectin, Collagen I, α-SMA, TK1, and PFKFB3 in mouse lung tissues (n=3), with * $P<0.05$, ** $P<0.01$ vs. the control group, and ##$P < 0.01$ vs. bleomycin group. (C-G) Concentration of IL-6, IL-1β, and TNF-α in BALF and lung tissues by ELISA (n = 4), with *$P < 0.05$ vs. the control group, #$P < 0.05$ vs. the bleomycin group. (H-J) Relative mRNA expression of MMP2, MMP7, and MMP8 in lung tissues (n = 4), with *$P < 0.05$, **$P < 0.01$ vs. the control group, and #$P < 0.05$, ##$P < 0.01$ vs. the bleomycin group. For A, C, D, E, F, G, H, I, and J, one-way ANOVA was used. For B, 2-way ANOVA was used. Data are presented as mean ± SD. Source data are provided as a Source data file.
