# Supplementary material for: Vitamin D attenuates the progression of pulmonary fibrosis via inhibiting thymidine kinase 1/PFKFB3-driven glycolysis
Source: J Transl Med. 2026 Apr 22;24:571. doi: 10.1186/s12967-025-07298-1 (PMC13104274; doi:10.1186/s12967-025-07298-1)
Supplement: Supplementary file 2 — Supplementary Material 2 [file 12967_2025_7298_MOESM2_ESM.docx]

Table S1. Sequences of primers for qRT-PCR.

| Gene Name | Forward primer(5'-3') | Reverse primer(5'-3') |
| --- | --- | --- |
| MMP2(Mus) | ACTCCGGAGATCTGCAAACA | ACTGTCCGCCAAATAAACCG |
| MMP7(Mus) | GAACTTCCTGTTTGCTGCCA | TCTGAATGCCTGCAATGTCG |
| MMP8(Mus) | CAGGCCAAGGTATTGGAGGA | ACATCAAGGCACCAGGATCA |
| GAPDH(Mus) | GTCAAGGCTGAGAACGGGAA | AAATGAGCCCCAGCCTTCTC |
| TK1(Homo) | GCCAAAGACACTCGCTACAG | GCCACAATTACGGTCTTCCC |
| PFKFB3(Homo) | TTGGCGTCCCCACAAAAGT | AGTTGTAGGAGCTGTACTGCTT |
| GAPDH(Homo) | GCATCCTGGGCTACACTG | TGGTCGTTGAGGGCAAT |

Table S2. Sequences of siRNA against VDR and TK1.

| Gene Name | siRNA target sequences |
| --- | --- |
| VDR | GGAUCUGAGUGAAGAAGAUTT |
| TK1 | ACAAGTGCCTGGTGATCAA |

Table S3. Source of primary and secondary antibodies used in this study.

| Antibody Type | Antibodies | Company | Cat. No | Dilution rate |
| --- | --- | --- | --- | --- |
| primary antibody | Fibronectin | Abcam | ab45688 | 1:10000 |
| primary antibody | Collagen I | Abcam | ab260043 | 1:1000 |
| primary antibody | Collagen I | Abcam, USA | ab34710 | 1:500 |
| primary antibody | Collagen I | Proteintech, China | 67288-1-Ig | 1:500 |
| primary antibody | Alpha-smooth muscle Actin(α-SMA） | Abcam | ab124964 | 1:10000 |
| primary antibody | Tk1//Thymidine Kinase 1 | Abclonal, China | A5612 | 1:1000 |
| primary antibody | PFKFB3 | ABclonal, China | A22317 | 1:1000 |
| primary antibody | CYP24A1 | ABclonal, China | A22615 | 1:200 |
| primary antibody | GAPDH | Proteintech, China | 10494-1-AP | 1:5000 |
| secondary antibody | HRP goat anti-rabbit IgG (H+L) | Beyotime, China | A0208 | 1:2000 |
| secondary antibody | Cy3-conjugated goat anti-rabbit IgG (H+L) | Beyotime, China | A0516 | 1:500 |
| secondary antibody | FITC-conjugated goat anti-rabbit IgG (H+L) | Beyotime, China | A0562 | 1:500 |
| secondary antibody | FITC-conjugated goat anti-mouse IgG (H+L) | Beyotime, China | A0568 | 1:500 |

Table S4. Chemicals, peptides, and recombinant proteins.

| Regent | Company | Cat. No |
| --- | --- | --- |
| Crystal Violet Staining Solution | Beyotime, China | C0121 |
| Bleomycin sulfate | MedChemExpress, USA | HY-17565 |
| Silicon dioxide | Sigma-Aldrich, USA | S5631 |
| Trizol reagent | Vazyme, China | R401-01-AA |
| Dulbecco’s modified Eagle’s medium (DMEM) | Life Technologies/Gibco, Grand Island, NY | 11965118 |
| Minimum Essential Medium | Life Technologies/Gibco, Grand Island, NY | 11095080 |
| fetal bovine serum | Vivacell | C04001-500 |
| Penicillin-Streptomycin Solution | Beyotime, China | C0222 |
| Vitamin D3 | GLPBIO, USA | GC13852 |
| 1,25(OH)_2_D_3_ | MedChemExpress, USA | HY-15398 |
| 3PO | MedChemExpress,USA | HY-19824 |
| 2-Deoxy-d-glucose | MedChemExpress, USA | HY-13966 |
| rat tail-derived type I collagen solution | Xinyou Biotechnology Co., Ltd. | 200110 |
| HisyGo All-in-One RT Red SuperMix for qPCR | Vazyme, China | R222-01-AB |
| ChamQ Universal SYBR qPCR Master Mix | Vazyme, China | Q711-02-AA |

Table S5. Critical commercial assays

| Regent | Company | Cat. No |
| --- | --- | --- |
| BCA Protein Assay kit | Beyotime, China | P0012 |
| ATP assay kit | Beyotime, China | S0026 |
| Lactic Acid assay kit | Jiancheng Bioengineering Insitute, China | A019-2-1 |
| glucose assay kit | BioVision, Milpitas, CA, USA | 20241106101 |
| Hydroxyproline content assay kit | Nanjing Jiancheng Bioengineering Insitute | A030-2-1 |
| Extracellular acidification rate assays | Elabscience, China | E-BC-F069 |
| EdU Cell Proliferation Kit | Beyotime, China | C0071S |
| MTT Cell Proliferation and Cytotoxicity Assay Kit | Beyotime, China | C0009S |
| 25-HVD3(25-Hydroxy Vitamin D3) ELISA Kit | Elabscience, China | E-EL-0015 |
| Mouse IL-6(Interleukin 6) ELISA Kit | ELK Biotechnology, China | ELK1157 |
| Mouse IL1-β1(Interleukin 1 Beta) ELISA Kit | ELK Biotechnology, China | ELK1271 |
| Mouse TNF-α（Tumor Necrosis Factor Alpha）ELISA Kit | ELK Biotechnology, China | ELK1387 |
